# Biodata-centric cardiovascular disease prediction using multi-objective genetic algorithm-driven deep ensembles

**DOI:** 10.1038/s41598-025-28771-3

**Published:** 2025-12-05

**Authors:** Magda M. Madbouly, Saad M. Darwish, Noha A. El-Shoafy

**Affiliations:** 1 Faculty of Computers and Data Science, Alexandria University, Alexandria, 5432042 Egypt; 2 Department of Information Technology, Institute of Graduate Studies and Research, Alexandria University, Alexandria, 21526 Egypt; 3 Faculty of Computers and Artificial Intelligence, Matrouh University, Matrouh, 51511 Egypt

**Keywords:** Biodata mining, Cardiovascular disease prediction, Multi-Objective genetic algorithm, Deep ensemble learning, Feature selection and optimization, Cardiology, Computational biology and bioinformatics, Diseases, Health care, Mathematics and computing

## Abstract

The increasing availability of patient BioData, including clinical measurements and physiological indicators, offers unprecedented opportunities for developing intelligent, data-driven diagnostic tools. In the context of cardiovascular disease (CVD)—the leading cause of mortality globally—mining such BioData effectively is critical for enabling early detection and supporting complex clinical decision-making. However, traditional predictive models often fail with inherent trade-offs, such as balancing predictive accuracy across imbalanced classes, minimizing feature redundancy, and ensuring model interpretability. To address these limitations, this study introduces a two-stage prediction framework for heart disease. First, a Multi-Objective Genetic Algorithm (MOGA) is employed to perform optimal feature selection by simultaneously maximizing classification accuracy and minimizing redundancy among the 13 clinical features of the UCI Cleveland Heart Disease dataset. Second, the selected features are used to train a deep ensemble of regularized Multi-Layer Perceptrons (MLPs). The ensemble outputs are aggregated using uniform weighting and further refined through AdaBoost-based fusion to enhance robustness. This integrated approach ensures that the model captures clinically meaningful patterns across diverse patient profiles, while also improving interpretability for medical practitioners. Experimental results demonstrate that the proposed framework achieves 96% accuracy, 97% sensitivity, and an AUC-ROC of 0.978, outperforming several baseline machine learning models. The findings confirm the potential of the proposed MOGA–MLP ensemble framework for real-world clinical deployment, offering a reliable, interpretable, and generalizable solution for cardiovascular risk prediction.

## Introduction

Cardiovascular disease (CVD) prediction is a critical area of medical research and healthcare due to its potential to prevent life-threatening conditions such as heart attacks and strokes. Early and accurate prediction of CVD enables timely intervention, reducing mortality rates and healthcare costs. The importance of CVD prediction extends across various applications including clinical decision support systems, personalized healthcare, remote patient monitoring, and public health planning. However, developing reliable prediction models is complex due to the presence of multiple, and sometimes conflicting, objectives—such as maximizing accuracy while ensuring interpretability, maintaining sensitivity without sacrificing specificity, and balancing precision with recall. These trade-offs pose significant challenges in designing robust and generalizable models, especially when applied across diverse populations and healthcare settings^1,2^.

Several critical factors influence the difficulty of CVD prediction, requiring careful consideration during model development. Key features include chest pain type (cp.), resting electrocardiographic results (restecg), exercise-induced angina (exang), ST depression induced by exercise relative to rest (oldpeak), number of major vessels colored by fluoroscopy (ca.), and the thalassemia condition (thal). Additional factors such as age, gender, blood pressure, cholesterol levels, and fasting blood sugar levels also play a vital role. These variables exhibit high interdependence and variability, complicating the modeling process, especially under uncertainty or in the presence of noisy data. Moreover, the imbalanced distribution of positive and negative cases, along with limited labeled data in real-world settings, further exacerbates the prediction problem, highlighting the need for advanced methods like ensemble learning, optimization algorithms, and uncertainty-aware models^3–6^.

Single-objective and multi-objective approaches to CVD prediction differ fundamentally in how they balance performance criteria during model development. Single-objective approaches focus on optimizing one specific metric—typically accuracy, sensitivity, or specificity—aiming to produce the best possible result for that single goal. While these models are simpler and computationally efficient, they often fail to address real-world clinical complexities, such as the trade-off between false positives and false negatives, which can have serious implications in medical decision-making. On the other hand, multi-objective approaches aim to simultaneously optimize two or more conflicting objectives—such as maximizing sensitivity while minimizing false positives or balancing accuracy with model interpretability. These approaches typically use techniques like Pareto optimization, evolutionary algorithms, or weighted scoring methods to find optimal trade-offs. Their advantages include better alignment with clinical priorities, improved generalization to diverse patient populations, and the ability to provide more flexible decision support. However, they also come with disadvantages, such as increased computational complexity, longer training times, and the need for careful tuning of trade-off parameters, which can make implementation and interpretation more challenging for practitioners^7–10^. Table 1 summarizes the trade-offs between the simplicity and focus of single-objective models versus the real-world applicability and robustness of multi-objective approaches in CVD prediction.

Multi-objective genetic algorithm (MOGA) approaches play a vital role in prediction by enabling the simultaneous optimization of multiple conflicting objectives. These algorithms simulate the process of natural selection to evolve a population of solutions over generations, using operations like selection, crossover, and mutation. The role of optimization in multi-objective decision-making is crucial, as it helps identify the best trade-offs among competing goals rather than forcing a single, potentially biased solution. Among various optimization strategies, Pareto optimization is particularly preferred because it does not require pre-defining weightings for each objective—instead, it seeks a set of non-dominated solutions known as the Pareto front, where no objective can be improved without worsening another. This approach offers a more comprehensive and fair evaluation of alternatives, giving decision-makers the flexibility to choose from multiple optimal solutions based on clinical priorities or resource constraints. Compared to scalarization methods or weighted sum approaches, Pareto optimization provides better coverage of the solution space and avoids issues like poor sensitivity to objective scaling or missing optimal regions due to fixed trade-off assumptions^11–13^.

The deep ensemble learning approach for prediction enhances the reliability, robustness, and generalization of traditional deep learning models by combining the predictive strengths of multiple independently trained deep networks. Rather than relying on a single model, which may be sensitive to noise, data imbalance, or overfitting, the ensemble approach aggregates the outputs of several models—such as different configurations of convolutional neural networks (CNNs), multilayer Perceptrons (MLPs), or recurrent networks—through methods like majority voting, averaging, or weighted fusion. This strategy reduces model variance and captures a broader representation of complex, nonlinear patterns within patient data. Compared to traditional deep learning, which may yield inconsistent results when exposed to slightly varied inputs or unseen data, the ensemble method offers superior prediction stability, higher accuracy, improved uncertainty estimation, and resilience against overfitting. Additionally, deep ensembles are particularly effective in handling class imbalance, a common issue in medical datasets, by diversifying model learning and enhancing minority class recognition. Though computationally more demanding, deep ensemble approaches provide a more trustworthy and clinically applicable solution for CVD prediction, especially in real-world healthcare environments where decision reliability is critical^14–16^.

**Table 1:** Comparison between single-objective and multi-objective approaches for CVD prediction.

Aspect	Single-objective CVD prediction	Multi-objective CVD prediction
Goal	Optimize one specific performance metric (e.g., accuracy).	Optimize multiple, often conflicting objectives (e.g., accuracy & recall).
Complexity	Simpler model structure and implementation.	More complex due to trade-off handling and multi-criteria evaluation.
Interpretability	Easier to interpret and explain.	May be harder to interpret due to involvement of multiple trade-offs.
Flexibility	Limited adaptability to diverse clinical needs.	Flexible and better suited for real-world clinical scenarios.
Performance trade-offs	Ignores trade-offs; may overfit to a single metric.	Handles trade-offs explicitly (e.g., Pareto front, weighted optimization).
Optimization methods	Traditional machine learning algorithms (e.g., SVM, logistic regression).	Evolutionary algorithms, Pareto optimization, weighted loss functions.
Computational requirements	Generally lower computational cost.	Higher due to simultaneous optimization of multiple objectives.
Clinical relevance	May miss critical aspects like false negative cost.	More aligned with clinical decision-making and risk balancing.
Use case suitability	Suitable for well-defined, narrow problems.	Preferred for complex, real-world healthcare applications.
Scalability to diverse data	Fail with heterogeneous data and variable feature importance.	More robust in handling diverse patient data and complex relationships.

### Contribution

This work introduces, for the first time in the literature, a novel linkage between MOGAs and ensemble deep learning in the context of CVD prediction—marking a shift from traditional hybrid techniques to a deeper semantic integration that improves both predictive performance and clinical interpretability. In this novel approach, the output of the MOGA—specifically, the optimized feature subsets selected through multi-objective criteria like accuracy, sensitivity, and feature relevance—serves as the input to the ensemble deep learning framework. This is not merely a pre-processing step; it is a semantic bridge where the MOGA’s context-aware, Pareto-optimal feature selection process shapes how the ensemble learning models perceive and learn from the data. The selected features are not arbitrary—they reflect the best trade-offs among competing clinical and data-driven priorities. Thus, the ensemble classifier is semantically enhanced, as it is trained on feature sets that are not only statistically relevant but also optimized to align with real-world medical objectives.

To implement this semantic link in a meaningful way, the fitness function of the MOGA is designed to consider not only traditional metrics (e.g., classification accuracy or F1-score) but also properties that influence ensemble performance, feature diversity, model compatibility, and class imbalance handling in our case. The selected feature subsets from the MOGA are then used to train multiple deep learning models, each tuned to different optimal subsets, enhancing diversity within the ensemble. These models can then be combined using fusion strategies (weighted voting), where the weights are derived from the MOGA’s performance scores. This ensures that each model contributes to the final decision based on how well it performs on its optimized feature set, creating a semantically aware, context-sensitive ensemble system. In essence, the semantic link creates a feedback loop, where the optimization process informs the learning process, and the learning process validates the utility of the optimization, resulting in a synergistic framework tailored to the complex and uncertain nature of CVD prediction.

This paper is structured as follows: The literature review is presented in Sect. 2 along with the relevant works. In Sect. 3, the offered MOGA-based ensemble learning classifier for CVD predication is detailed. Section 4 discusses the experimental results. Lastly, Sect. 4 presents conclusions and plans for further research.

## Literature review

In recent years, significant advancements have been made in utilizing machine learning (ML) for heart disease classification, drawing from both widely-used open-access datasets like the UCI Cleveland dataset and confidential clinical records. Researchers have demonstrated that ML models, particularly tree-based algorithms and deep neural networks, consistently outperform traditional statistical methods in predicting heart-related conditions such as myocardial infarction and coronary artery disease. As highlighted by Bharti et al.^17^, the superiority of these models lies in their ability to process high-dimensional medical data and to automatically identify intricate, nonlinear patterns and relationships among clinical features—patterns that conventional techniques often fail to detect. This capability enables ML systems to deliver more accurate, reliable predictions, making them valuable tools for early diagnosis and risk assessment in clinical practice.

Heart disease datasets often encompass a wide array of clinical and demographic attributes, including blood pressure readings, cholesterol levels, electrocardiogram (ECG) measurements, age, gender, and other physiological indicators^18^. While this wealth of information can be valuable, not all features contribute meaningfully to the predictive modeling process. In fact, the presence of irrelevant or redundant attributes can introduce noise, inflate model variance, and lead to overfitting—ultimately degrading the model’s generalization ability. To address these challenges, researchers have increasingly turned to feature selection techniques that help identify and retain only the most informative predictors. For example, Biswas et al.^19^ systematically assessed the performance of various feature selection methods, such as Mutual Information (MI), Analysis of Variance (ANOVA), and the Chi-Square test, in combination with different machine learning classifiers. Their findings revealed that MI was particularly effective, especially when paired with sophisticated models like Random Forest, resulting in notable improvements in prediction accuracy and training efficiency. Likewise, Elmi et al.^20^ applied advanced feature selection strategies to enhance both the precision and interpretability of cardiovascular disease prediction systems. Their work underlined the necessity of integrating feature selection within machine learning pipelines to optimize outcomes. Given that real-world medical datasets may include dozens or even hundreds of potential predictors, it becomes imperative to employ robust computational approaches capable of isolating the features most closely associated with the onset and progression of heart conditions.

Deep Neural Networks (DNNs) have shown considerable potential in detecting complex, non-linear relationships within medical datasets, particularly when trained on large-scale patient data. In a 2024 study, Hu et al.^21^ developed an advanced deep learning-based early warning system for cardiovascular disease that effectively combined convolutional and recurrent neural layers. This hybrid architecture was designed to extract both spatial patterns from ECG signals and temporal trends from clinical records, resulting in enhanced prediction accuracy compared to conventional models. This approach reflects a broader movement in the medical AI community toward using deep learning architectures capable of integrating multimodal health data for more accurate early diagnoses. Despite their strengths, the real-world application of DNNs in clinical environments faces several limitations, including the lack of well-annotated datasets, class imbalance, and a high risk of overfitting—problems that are especially dominant in healthcare contexts. To overcome these barriers, researchers are increasingly adopting a combination of deep learning, feature selection, and metaheuristic optimization methods. For instance, Al-Mahdi et al.^22^ suggested a hybrid framework that employs Genetic Algorithms (GA) for selecting the most relevant features, which are then processed by a deep learning model further optimized using the Tunicate Swarm Algorithm (TSA). This integrated strategy significantly enhanced the accuracy and robustness of heart disease classification, demonstrating how intelligent feature selection and optimization can effectively mitigate the challenges posed by high-dimensional and redundant clinical data.

Ensemble learning has simultaneously gained prominence as a powerful approach for improving prediction accuracy, robustness, and model generalization, particularly in sensitive applications like medical diagnosis. Unlike traditional single-model strategies that may suffer from overfitting or dataset-specific biases, ensemble methods combine the predictions of multiple base classifiers to produce more balanced and reliable outcomes. This is especially important in clinical settings, where the inherent variability in patient data and the limited availability of labeled samples can compromise the performance of individual models. A notable example is the HeartEnsembleNet model introduced by Zaidi S. et al.^23^, which integrated several machine learning classifiers in conjunction with feature selection techniques to predict cardiovascular disease risk. This ensemble achieved a high accuracy of 92.95%, demonstrating how diversity among base learners can effectively reduce variance and enhance predictive stability across heterogeneous datasets. Similarly, the work of Al-Mahdi et al.^22^ also exemplifies an ensemble-based methodology, wherein multiple deep learning models are jointly optimized using metaheuristic techniques to outperform any single constituent model. These ensemble strategies are particularly well-suited to healthcare applications, as they provide the adaptability and comprehensiveness needed to handle diverse data types and complex clinical patterns, ultimately leading to more dependable and accurate diagnostic tools.

Interpretability remains a critical challenge in the deployment of machine learning systems within clinical environments, particularly in high-stakes fields such as cardiology. Tjoa and Guan^24^ underscore that the trustworthiness of AI-driven diagnostic tools is deeply tied to their transparency—clinicians and healthcare providers are more likely to embrace such systems when the reasoning behind predictions is clearly understandable. This issue persists even when a model demonstrates high predictive accuracy, as the absence of interpretability can lead to skepticism and underutilization in real-world medical settings. To address this, early developments in Explainable Artificial Intelligence (XAI) have introduced techniques such as feature importance scoring, Local Interpretable Model-Agnostic Explanations (LIME), and Shapley Additive Explanations (SHAP), which aim to clarify how input features contribute to a model’s output. These methods offer localized or global explanations for model decisions, but they often fall short of fully elucidating the internal workings of complex deep learning and ensemble systems. As pointed out in recent studies^25,26^, effectively combining XAI methodologies with ensemble learning frameworks remains a developing area of research. This integration is particularly relevant in cardiac diagnostics, where diverse patient data and intricate feature interactions require not only high-performance models but also interpretable outputs that can guide clinical decision-making with confidence.

Genetic Algorithms (GAs) have gained substantial traction in the domain of medical classification, particularly for optimizing both model hyperparameters and feature selection processes^22,27^. These biologically inspired metaheuristic algorithms simulate the process of natural selection to iteratively search for optimal or near-optimal solutions within complex search spaces. In the context of heart disease prediction, GAs are especially valuable for reducing the dimensionality of datasets by identifying and eliminating irrelevant or redundant features, thereby improving computational efficiency and model performance. For instance, Al-Mahdi et al.^22^ demonstrated that employing a GA to refine the feature subset and simultaneously tune the parameters of an ensemble learning model significantly enhanced classification accuracy on the UCI Cleveland dataset, achieving performance levels exceeding 97%. This improvement was attributed to the algorithm’s ability to identify the most informative features and optimal configuration settings, which together boosted model precision and generalizability. However, despite these advantages, many GA-driven optimization frameworks in medical AI research have predominantly emphasized a single performance metric—typically overall accuracy. This narrow focus can inadvertently overlook other clinically critical evaluation metrics, such as sensitivity (true positive rate), specificity (true negative rate), or cost-related implications of misclassification. As such, there is a growing recognition of the need for multi-objective optimization approaches that balance predictive performance with practical and ethical considerations relevant to real-world clinical decision-making.

Given the high-stakes nature of healthcare decision-making, where trade-offs between metrics like false positives and false negatives can significantly impact patient outcomes, researchers have increasingly turned to MOGAs to optimize machine learning models more holistically. Unlike traditional single-objective Genetic Algorithms that focus solely on maximizing a single performance metric—typically overall accuracy—MOGAs are designed to simultaneously optimize multiple, often conflicting, objectives. This approach generates a Pareto front of optimal solutions, each representing a different trade-off scenario among key performance metrics such as sensitivity, specificity, precision, and computational efficiency. Such a framework allows clinicians to choose the most appropriate model configuration based on specific clinical requirements, such as prioritizing high sensitivity to reduce the risk of missed diagnoses or enhancing specificity to minimize unnecessary interventions. A notable example is a study that implemented a MOGA-optimized deep learning model for medical image classification, where the algorithm concurrently tuned for both sensitivity and specificity, ultimately achieving a sensitivity of 98.2% and a specificity of 92.2%^28^. These results illustrate the value of MOGAs in developing flexible, context-aware diagnostic tools that align better with real-world healthcare priorities. By offering a spectrum of balanced solutions rather than a single optimized outcome, MOGA-based frameworks empower healthcare practitioners to make more informed, tailored decisions in diagnostic and prognostic applications.

Several recent studies highlight the effectiveness of multi-objective optimization techniques in advancing cardiovascular risk prediction models by addressing multiple critical performance metrics simultaneously. For example, Shamrat et al.^29^ developed an explainable, multi-objective hybrid machine learning framework that combines the Non-dominated Sorting Genetic Algorithm II (NSGA-II) for feature selection with a stacked ensemble classifier. This integrated approach not only optimized accuracy and sensitivity but also considered model complexity, striking a crucial balance that enhances the clinical applicability and interpretability of the heart failure prediction system. By managing these competing objectives, their model achieved more reliable and practical performance, better aligning with the nuanced demands of healthcare settings. Similarly, Ganie et al.^30^ applied a boosting ensemble model combined with an evolutionary optimization strategy that explicitly prioritized both sensitivity and specificity, key indicators in medical diagnostics. Their results demonstrated that this dual-focus optimization improved predictive performance, particularly in heterogeneous patient datasets where variations in demographics and clinical presentations pose challenges for standard modeling approaches.

Building on these advances, recent studies have further explored diverse optimization and ensemble strategies for cardiovascular risk prediction, highlighting both their potential and remaining limitations. Srikanth Kumar & Rekha^31^ applied a Gaussian-optimized dense neural network for heart disease prediction, achieving high accuracy but with limited generalizability. Kumar et al.^32^ proposed a hawk’s optimizer–driven ensemble that improved robustness at the cost of higher complexity. Arunachalam et al.^33^ employed ensemble classifiers like X-Boost and AdaBoost, showing adaptability but constrained by small datasets. Beyond UCI data, Saranya et al.^34^ developed a DenseNet-ABiLSTM hybrid for arrhythmia detection using PPG signals, capturing spatio-temporal features though facing interpretability issues. Rajagopal & Ranganathan^35^ assessed unsupervised dimensionality reduction, improving efficiency but risking loss of discriminative power. Together, these works trace the shift from optimization-driven feature selection to deep hybrid models, while revealing common challenges in scalability, interpretability, and dataset diversity. Our proposed framework directly addresses these gaps through a multi-objective optimization–guided ensemble approach.

Despite significant progress in heart disease prediction research, several critical challenges remain unresolved. One major limitation stems from data constraints—many studies rely on relatively small sample sizes, and the quality of available datasets often varies due to inconsistent data collection protocols and missing values. These factors can substantially impede the effectiveness of deep learning models, which typically require large, high-quality datasets to capture complex patterns reliably. Furthermore, there is a notable lack of external validation using diverse populations from multiple geographic regions or healthcare systems, which raises serious concerns about the models’ ability to generalize beyond the original study cohorts^36,37^. Another critical area that demands attention is interpretability. While MOGAs can optimize models across several performance criteria simultaneously, generating a Pareto front of optimal solutions, the final decision-making models often remain opaque to clinicians. For these tools to gain widespread acceptance in clinical settings—where trust in model predictions can directly impact patient care—there must be clear, transparent explanations of how and why specific predictions are made. The increasing integration of MOGA-based ensemble deep learning approaches with Explainable Artificial Intelligence (XAI) techniques is paving the way for prediction systems that not only achieve higher accuracy and balance multiple clinical objectives but also provide greater transparency and comprehensibility.

### Research gap

This work introduces an innovative semantic linkage between MOGAs and ensemble deep learning that transcends the traditional cascading approach—where feature selection and model training are sequential but disconnected processes—by deeply integrating the two into a unified framework for CVD prediction. Instead of simply passing MOGA-optimized feature subsets as a preliminary step to the ensemble models, our method utilizes these Pareto-optimal features—carefully balanced across clinical and statistical objectives such as accuracy, sensitivity, and relevance—to directly influence and guide the training of diverse deep learning classifiers within the ensemble. This semantic link transforms the relationship from a linear sequence into an interdependent process where the MOGA’s fitness function is explicitly designed to optimize not only classical metrics but also ensemble-specific factors like feature diversity, model synergy, and class imbalance management. Consequently, the ensemble aggregates these specialized models through weighted voting schemes, with weights derived from the MOGA’s multi-criteria performance, establishing a feedback loop that continually refines both optimization and learning stages. This tightly coupled, semantically aware framework goes beyond conventional cascading by embedding multi-objective optimization within the core of ensemble learning, offering a novel, synergistic solution tailored to the complex and uncertain nature of CVD prediction.

## Methodology

**Fig. 1:**
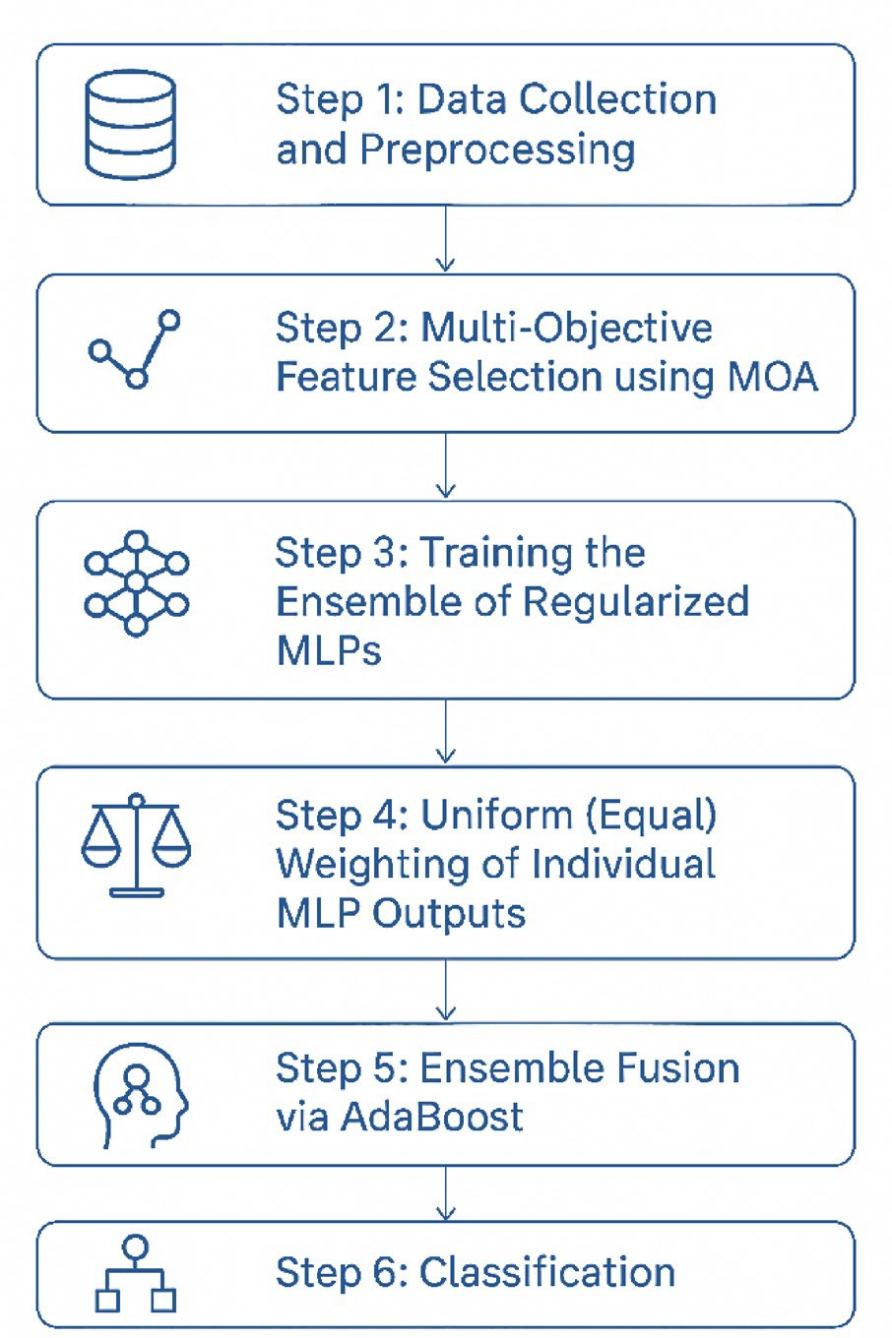
The suggested classification model for heart disease prediction.

The proposed framework achieves heart disease prediction through the semantic integration of MOGA and MLPs, where MOGA-driven feature selection directly informs the learning of deep ensemble classifiers. First, the UCI Cleveland Heart Disease dataset is preprocessed by handling missing values, applying one-hot encoding to categorical features, and normalizing continuous attributes. Next, MOGA identifies a compact and clinically meaningful subset of features by optimizing both classification accuracy and feature redundancy. These selected features are then used to train an ensemble of regularized MLPs, with diversity introduced through different initializations and data splits. Finally, ensemble predictions are aggregated by uniform averaging and refined via AdaBoost-based fusion to adaptively strengthen weak learners. This integrated design ensures that feature relevance and classifier learning are jointly optimized, thereby enhancing predictive accuracy, reducing redundancy, and improving interpretability for clinical application. The following subsections outline the proposed model, detailing each stage to convey its design and functionality clearly with Fig. 1 illustrating the overall process.

### Step 1: data collection and preprocessing

The development of a reliable heart disease prediction system begins with acquiring and carefully preprocessing a comprehensive dataset. In this study, the UCI Heart Disease dataset is utilized (https://archive.ics.uci.edu/dataset/45/heart+disease), which is widely recognized for its robustness and diverse clinical variables. The dataset comprises 303 patient records collected from multiple clinical sources. These records include both categorical and continuous variables representing demographic data, clinical test results, and symptom-related information. Each entry is labeled to indicate the presence or absence of heart disease, forming a binary classification problem. The dataset includes 13 primary features: age, sex, chest pain type (cp.), resting blood pressure (trestbps), serum cholesterol (chol), fasting blood sugar (fbs), resting electrocardiographic results (restecg), maximum heart rate achieved (thalach), exercise-induced angina (exang), ST depression induced by exercise relative to rest (oldpeak), the slope of the peak exercise ST segment (slope), number of major vessels colored by fluoroscopy (ca.), and thalassemia (thal). The target variable (target) takes a value of 1 for the presence of heart disease and 0 for its absence.

To account for the diversity of the data, distinct preprocessing approaches were applied to categorical and continuous variables. Continuous features (e.g., age, trestbps, chol, thalach, oldpeak) often vary in scale and distribution, which may bias distance-based algorithms or affect convergence in neural networks. To address this, normalization technique using Min–Max scaling was tested to transform the features into comparable ranges. Categorical variables (e.g., sex, cp., restecg, exang, slope, ca., thal) represent diverse non-numeric clinical attributes. To preserve their semantic meaning while making them suitable for machine learning algorithms, one-hot encoding was applied. For example, chest pain type (cp.), which has four possible categories, was expanded into four binary columns, ensuring that no ordinal bias was introduced.

Handling missing values was another essential step, particularly in the ‘ca’ and ‘thal’ attributes, where incomplete records were present. Instead of discarding samples, which could reduce the dataset’s diversity and predictive strength, imputation techniques were employed. Numerical features with missing values were imputed using mean substitution, while categorical features were imputed using mode substitution. This ensured minimal information loss while maintaining consistency across the dataset. Finally, class balance and representativeness of the data were verified to ensure the dataset did not disproportionately favor one class (presence vs. absence of heart disease). To achieve this, the distribution of the target variable was carefully examined. Specifically, the frequency of patients with heart disease (target = 1) and without heart disease (target = 0) was calculated and visualized using histograms and bar plots. This allowed us to detect whether the dataset exhibited significant imbalance that could bias the learning process. In the UCI Heart Disease dataset, the classes are relatively balanced, with 165 samples labeled as presence of heart disease and 138 labeled as absence. This near-equilibrium minimizes the risk of classifier bias toward the majority class.

In addition to class balance, representativeness of feature diversity was verified by exploring the distribution of categorical and continuous attributes. For categorical variables such as chest pain type (cp.), slope, and thal, frequency tables were used to ensure that all categories were represented with sufficient occurrences. For continuous features like age, cholesterol (chol), and maximum heart rate (thalach), descriptive statistics (mean, standard deviation, min, max) and boxplots were analyzed to confirm reasonable variability and the absence of extreme skewness or data anomalies. This multi-step verification process ensured that the dataset retained both balance and diversity, thereby reducing the risk of biased or overfitted models in subsequent predictive tasks^7,8,10,31,32^.

Given the relatively small size of the UCI Heart Disease dataset, data augmentation techniques were incorporated to enhance the effective sample size and model robustness. Specifically, the Synthetic Minority Over-sampling Technique (SMOTE) was applied to generate synthetic samples for both classes, particularly focusing on preserving the diversity of feature values in minority or underrepresented patterns. This augmentation not only helps maintain class balance but also mitigates overfitting by providing additional representative samples for model training, thereby improving the generalization performance of the predictive models^2^.

Before applying multi-objective feature selection, we extracted statistical descriptors from the raw clinical features to enhance the representational quality of the dataset. Each numerical attribute (e.g., cholesterol, maximum heart rate, resting blood pressure, ST depression) was transformed into additional statistical features such as mean, standard deviation, minimum, maximum, and interquartile range computed across stratified patient groups. These statistical descriptors capture distributional characteristics that are not always evident from single raw values. For categorical variables (e.g., chest pain type, thalassemia, resting ECG), frequency-based encoding and conditional probability distributions were employed to derive informative statistical summaries. This transformation allowed the model to control both direct clinical measurements and their statistical behavior across the population, ensuring a richer and more discriminative feature space for subsequent optimization. By incorporating these statistical descriptors, the feature space not only retained clinically meaningful attributes but also included higher-level statistical patterns that improve class separability. These extracted features were then used as input to the MOGA framework, ensuring that the optimization process considered both original and derived statistical information when identifying optimal feature subsets.

### Step 2: Multi-objective feature selection using MOGA

In the second phase, the goal is to identify the most optimal subset of features from the preprocessed heart disease dataset, balancing competing objectives through a MOGA. Rather than simply reducing dimensionality, MOGA strategically navigates complex trade-offs to select features that are not only statistically robust but also clinically relevant. The algorithm operates on the refined dataset obtained from Step 1, which contains 13 distinct clinical features. These features vary in informativeness, correlation, and interpretability, and selecting the right combination is critical for improving the overall performance and generalization of the learning models^11–13^.

MOGA handles key conflicting objectives during feature selection. For instance, it evaluates the trade-off between relevance and redundancy, such as when two features like “cholesterol” and “oldpeak” contribute similarly to prediction but may carry overlapping information. Instead of including both and risking overfitting, MOGA attempts to retain only the more informative one or find a balanced representation. Similarly, the algorithm manages the balance between interpretability and predictive power. While variables like “thal” may be statistically significant, their abstract encoded forms (e.g., numerical codes for defect types) may not translate easily into clinical insights. The algorithm weighs such cases to ensure the selected features not only optimize performance but remain actionable for clinical professionals.

Furthermore, MOGA effectively addresses the inherent trade-off between maximizing model accuracy and maintaining sensitivity to class imbalance. In classification problems—especially those involving imbalanced datasets—optimizing solely for accuracy can lead to biased models that favor the majority class while neglecting the minority class. MOGA approaches this challenge by treating accuracy and class imbalance sensitivity as separate, often conflicting objectives. By evolving a population of solutions through genetic operations, it seeks an optimal balance where the model achieves high accuracy without sacrificing its ability to detect underrepresented classes. It also evaluates the tension between clinical validity and data-driven discovery, where statistically powerful features might not align with established medical knowledge. Through Pareto optimization, the algorithm ensures that such features are carefully considered within the broader objective landscape, maintaining both statistical precision and domain relevance^28,29^.

To implement this Pareto-based optimization, MOGA utilizes a fitness function that evaluates each candidate feature subset based on multiple objectives simultaneously. The equation is formulated as follows:

1 $$\:Fitness=\left[{f}_{1}\right(S),{f}_{2}(S),{f}_{3}(S),{f}_{4}(S\left)\right].$$

The proposed model evaluates a candidate feature subset $\:\:S$using four distinct objective functions, each capturing a critical aspect of performance and robustness:

- $\:{\varvec{f}}_{1}\left(\varvec{S}\right)$: **Classification Accuracy**: This objective quantifies how well the selected feature subset contributes to the overall predictive performance of the classifier. It is typically measured using metrics such as accuracy, F1-score, or AUC, depending on the task (accuracy in our case). A higher value indicates that the subset provides strong discriminative information for accurate class predictions.
  2 $$\:{f}_{1}\left(S\right)=\frac{TP+TN}{TP+TN+FP+FN}$$
  This function provides a direct measure of the proportion of correct predictions out of all predictions, serving as a key indicator of the model’s overall effectiveness.
- $\:{\varvec{f}}_{2}\left(\varvec{S}\right)$: **Redundancy Penalty**: This function penalizes the presence of redundant or highly correlated features within subset$\:\:S$. Redundancy is often measured using pairwise correlation coefficients or mutual information between features (pairwise Pearson correlation in our case). Reducing redundancy enhances model efficiency and interpretability by eliminating overlapping information that does not contribute to additional predictive power.
  3 $$\:{f}_{2}\left(S\right)=\frac{1}{\left|S\right|\left(\left|S\right|-1\right)}\sum\:_{i,j\in\:S\:,\:i\ne\:j}\left|corr\left(i,j\right)\right|$$
  $\:\left|S\right|$ is the cardinality of feature subset, this is the number of features selected in subset $\:S$. $\:corr(i,j)\:$is the Pearson Correlation. The result is a value between − 1 and + 1.
- $\:{\varvec{f}}_{3}\left(\varvec{S}\right)$: **Interpretability Score**: Interpretability reflects how understandable and actionable the selected features are from a domain-expert perspective. This score can be derived based on prior domain knowledge, or semantic relevance. A higher interpretability score indicates that the subset consists of simpler, more meaningful features that are easier for users to comprehend and validate.
  4 $$\:{f}_{3}\left(S\right)=\frac{1}{\left|S\right|}\sum\:_{i\in\:S}\text{}interpretability\left(i\right)$$
  Assign interpretability scores manually (e.g., 3 = high, 2 = medium, 1 = low), then compute average. Interpretability scores are assigned based on clinical relevance and complexity: features like age, sex, chol, and trestbps receive a score of 3 for being straightforward and widely recognized by clinicians; moderately interpretable features such as thal, ca., and ST slope are scored 2; while more abstract or transformed features, including encoded values of thal or resting ECG results (restecg), are given a score of 1 due to their reduced clinical transparency.
- $\:{\varvec{f}}_{4}\left(\varvec{S}\right)$: **Sensitivity to Class Imbalance**: This objective measures the model’s ability to perform well across both majority and minority classes when trained using the feature subset$\:\:S$. It is often quantified using metrics such as the geometric mean (G-mean) of sensitivity (recall) across all classes (G-mean in our case). A higher value indicates that the subset supports balanced performance and mitigates the bias typically introduced by imbalanced datasets.
  5 $$\:{f}_{4}\left(S\right)=\sqrt{Sensitivity\times\:Specificity}$$
  6 $$\:Sensitivity\:=\frac{TP}{TP+FN}$$
  7 $$\:\:Specificity\:=\:\frac{TN}{TN+FP}$$

This measure ranges from 0 to 1, where 1 indicates perfect classification of both classes, and values closer to 0 indicate poor performance on at least one class. For example, in a heart disease dataset with 90% healthy (negative) and 10% disease (positive), a model that always predicts “healthy” might have high accuracy but a G-Mean of 0, since sensitivity would be 0 (it missed all positive cases). G-Mean helps ensure the model does not ignore the minority class, making it ideal for imbalanced clinical data where detecting the positive class (e.g., heart disease presence) is crucial.

Each solution (subset of features$\:\:S$) is represented as a binary chromosome, where 1 indicates inclusion of a feature and 0 indicates exclusion. Example chromosome: [1, 0, 1, 0, 1, 0, 0, 1, 1, 0, 0, 0, 1] → Selected features: age, cp., chol, thalach, exang, thal. The MOGA evolves these chromosomes over multiple generations using selection, crossover, and mutation operators, and identifies a Pareto front of non-dominated solutions. Each point on this front represents a unique trade-off between competing objectives, giving downstream ensemble models a semantically optimized space in which to learn and collaborate. The final output of the model is a Pareto front consisting of multiple candidate feature subsets, each representing a different balance among competing objectives such as accuracy, redundancy, interpretability, and sensitivity to class imbalance. Users can select the most appropriate subset based on their specific preferences—for instance, prioritizing high accuracy along with moderate interpretability. Example Output (Subset 1) includes the features cp., thalach, exang, oldpeak, ca., and thal, achieving an accuracy of 87%, a redundancy score of 0.12, an interpretability score of 1.8, and a G-Mean of 0.83, indicating a well-balanced subset in terms of predictive performance and generalizability across imbalanced classes.

Algorithm 1 outlines the pseudocode for the MOGA-based feature selection process, designed to generate a Pareto front of optimal feature subsets by simultaneously optimizing multiple conflicting objectives such as accuracy, redundancy, interpretability, and class imbalance sensitivity. Through evolutionary operations like selection, crossover, and mutation, the algorithm iteratively refines the population to identify feature combinations that best balance these trade-offs^11–13,28,29^ (Algorithm 2).

**Algorithm 1:**
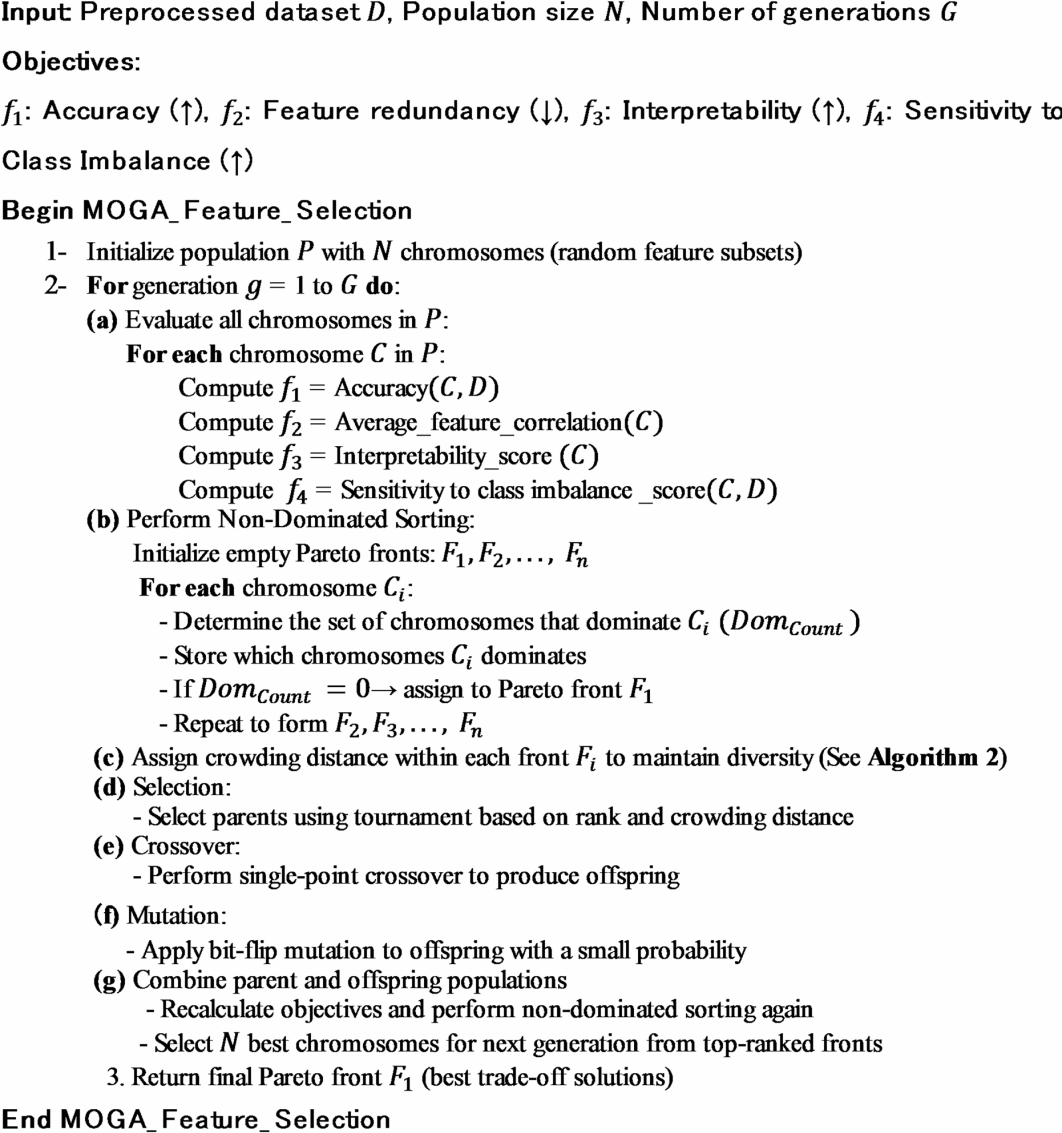
MOGA pseudocode for Pareto front feature selection.

**Algorithm 2:**
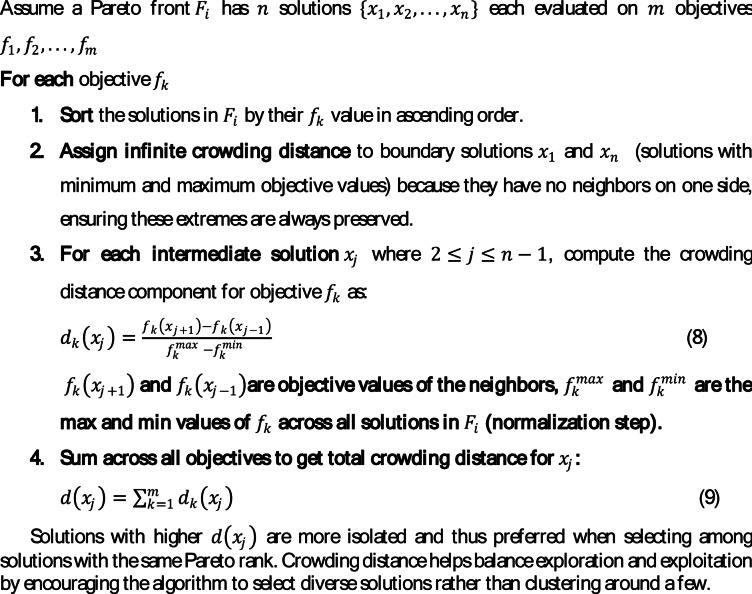
Crowding distance computation.

### Step 3: training the ensemble of regularized MLPs

Based on the MOGA using Pareto-based optimization, four distinct feature subsets were extracted from the UCI heart disease dataset, each emphasizing a specific objective. The Accuracy-optimized subset includes cp. (chest pain type), thalach (maximum heart rate), oldpeak (ST depression), exang (exercise-induced angina), ca. (number of vessels), and thal (thalassemia), achieving the highest predictive performance with 87% accuracy, while maintaining acceptable interpretability. The Redundancy-minimized subset focuses on uncorrelated features and includes age, sex, fbs (fasting blood sugar), restecg (resting ECG results), slope (slope of ST segment), and trestbps (resting blood pressure), resulting in a very low redundancy score of 0.06, favoring model simplicity and minimizing overfitting due to feature overlap. The Interpretability-optimized subset prioritizes features easily understood by clinicians, comprising age, sex, chol (serum cholesterol), trestbps, cp., and exang, leading to a high interpretability score of 2.8, ideal for clinical deployment and decision support. Lastly, the Imbalance-sensitive subset, tailored for maximizing robustness across skewed class distributions, includes thal, ca., oldpeak, cp., thalach, and slope, attaining a G-Mean of 0.86, indicating effective performance across both majority and minority classes. These four subsets on the Pareto front enable users to select the most suitable feature configuration depending on their priorities—be it predictive strength, simplicity, clinical relevance, or resilience to class imbalance—for subsequent training of ensemble models.

By training a Multi-Layer Perceptron (MLP) model on each distinct subset, the ensemble captures diverse semantic representations of the problem space. This semantic alignment between model and data ensures that the ensemble is not merely a collection of learners but a diverse panel of domain-informed specialists. Overall, training regularized MLPs on MOGA-derived feature subsets creates a rich ensemble that is both data-efficient and semantically grounded, yielding stronger and more trustworthy predictions^15–17,21^.

#### Input layer configuration

Each MLP receives as input one of the four MOGA-derived subsets, each containing 6 features. Consistent input size ensures architectural uniformity and fair performance comparison across MLPs.

10 $$\:Input\:Size\:\left({n}_{in}\right)=6$$

- MLP₁ (Accuracy): cp., thalach, oldpeak, exang, ca., thal.
- MLP₂ (Redundancy): age, sex, fbs, restecg, slope, trestbps.
- MLP₃ (Interpretability): age, sex, chol, trestbps, cp., exang.
- MLP₄ (Imbalance): thal, ca., oldpeak, cp., thalach, slope.

#### Hidden layer design

Each MLP has 2 hidden layers, sized based on the input dimension, Layer 1: $\:{n}_{1}=2\times\:{n}_{in}=12\:$, Layer 2: $\:{n}_{2}={n}_{in}=6$ with Activation Function (ReLU):

11 $$\:{a}_{j}^{\left(l\right)}=\varnothing\:\left(\sum\:_{i=1}^{{n}_{l}-1}{\omega\:}_{ji}^{\left(l\right)}.{a}_{i}^{(l-1)}+{b}_{j}^{\left(l\right)}\right)$$

$\:\varnothing\:(.)$ is the ReLU activation function $\:\varnothing\:\left(x\right)=\text{m}\text{a}\text{x}(0,x)$, $\:{\omega\:}_{ji}^{\left(l\right)}$ is the weight from neuron $\:i$ in $\:l-1$ layer to neuron$\:\:j$ in layer$\:\:l$, and $\:{b}_{j}^{\left(l\right)}$ is the bias term. This Provides sufficient capacity to learn nonlinear interactions without overfitting small tabular data. the bias term $\:{b}_{j}^{\left(l\right)}$ acts as an affine shift that allows each neuron to learn patterns even when input values are zero or centered. In the context of the UCI Heart Disease dataset, bias values are not derived directly from the dataset but are learned parameters initialized (typically using zero, small constants, or random values from a normal or uniform distribution) and optimized through backpropagation. During training, for each selected feature subset, the model updates the bias terms by computing the gradient of the loss function with respect to the biases using chain rule. The optimization algorithm (e.g., Adam) then adjusts $\:{b}_{j}^{\left(l\right)}$ iteratively to minimize the total loss, including the binary cross-entropy and regularization components. Thus, biases are adaptively tuned to fit the specific feature characteristics and statistical distributions of the selected UCI dataset subset, contributing significantly to the expressiveness and accuracy of the neural model.

#### Output layer configuration

Output neurons is 1 with sigmoid activation function that is Suitable for binary classification, output interpretable as class probability.

12 $$\:\widehat{y}\text{}\:=\sigma\:\left(z\right)=\frac{1}{1+{e}^{-z}}\:,\:\:\:\:\:\:z={\omega\:}^{T}x+b\:$$

$\:\widehat{y}$​ is the predicted probability that a given input $\:x$ belongs to the positive class (i.e., presence of heart disease), $\:\sigma\:\left(z\right)$ is the sigmoid activation function, which squashes any real-valued input $\:z$ into the range (0, 1), making it interpretable as a probability. $\:\omega\:$ is the weight vector of the output neuron, where each component $\:{\omega\:}_{i}$ represents the learned importance of the corresponding input feature $\:{x}_{i}$​, $\:x$ is the input feature vector, a numeric representation of patient attributes from the UCI dataset (e.g., cp., thalach, chol, etc.), $\:b$ is the bias scalar of the output neuron, allowing flexibility in the model’s decision threshold.

#### Loss and regularization

Controls overfitting by penalizing large weights and introducing stochasticity.

- Binary Cross-Entropy Loss:
  13 $$\:L\left(y,\widehat{y}\right)=-\left[y\text{log}\left(\widehat{y}\right)+\left(1-y\right)\text{l}\text{o}\text{g}(1-\widehat{y})\right]$$
- L2 Regularization: $$\:(\lambda\:\:=\:0.001$$)
  14 $$\:{L}_{reg}=L+\lambda\:\sum\:_{l}\sum\:_{j,i}{\left({\omega\:}_{ji}^{\left(l\right)}\right)}^{2}$$

Dropout: 30% (rate = 0.3) after each hidden layer. $\:L$ is the original loss function (e.g., binary cross-entropy) that measures how well the model’s predictions match the actual labels. For classification tasks, it penalizes incorrect predictions. $\:\lambda\:$ is the regularization coefficient (hyperparameter), which controls the strength of the penalty. A higher $\:\lambda\:\:$increases regularization, reducing model complexity but possibly underfitting.

### Step 4: uniform (equal) weighting of individual MLP outputs

At this stage, the input to the uniform weighting module consists of four independent prediction outputs, each generated by one of the specialized MLPs trained on distinct MOGA-derived feature subsets (Accuracy-optimized, Redundancy-minimized, Interpretability-optimized, and Imbalance-sensitive). Each MLP outputs a probability score,$\:{\widehat{y}}_{k}\in\:\left(\text{0,1}\right)$, indicating the model’s confidence that a given patient has heart disease. These probability scores are the result of passing the input features through their respective trained architectures, including the hidden layers and sigmoid-activated output layer. Thus, the ensemble receives a 4-dimensional vector of predictions for each input sample, one from each domain-informed model. The output of this step is a single ensemble prediction,$\:{\widehat{y}}_{\text{e}\text{n}\text{s}\text{e}\text{m}\text{b}\text{l}\text{e}}$, computed as the arithmetic mean of the four individual MLP outputs. Mathematically, this can be expressed as:

15 $$\:{\widehat{y}}_{\text{e}\text{n}\text{s}\text{e}\text{m}\text{b}\text{l}\text{e}}=\frac{1}{4}\sum\:_{k=1}^{4}{\widehat{y}}_{k}$$

This output retains the probabilistic interpretation, as it remains bounded within (0, 1), and can be directly thresholded (e.g., at 0.5) for binary decision-making. Importantly, each MLP is treated equally in this aggregation step—no one model is prioritized over another—ensuring that the ensemble’s output is not biased toward any specific objective, such as accuracy or interpretability, from the start.

The use of uniform weighting in this context serves several key roles. First, it ensures a neutral consensus mechanism, particularly valuable in the early stages of ensemble design. Since each MLP was trained on a distinct subset optimized for a different objective, assigning equal weight prevents premature favoritism towards any particular feature perspective. This fusion supports a more holistic generalization, using strengths from all models without injecting bias based on heuristics or external assumptions. Second, uniform weighting acts as a robust regularizer for ensemble behavior. By balancing the influence of each MLP, it mitigates the risk of overfitting to one type of feature selection (e.g., a subset that over performs on training but generalizes poorly). This helps stabilize predictions across varied patient profiles and class distributions, particularly given the sensitivity of clinical data to bias and overfitting. In effect, it treats the ensemble as a committee of equals, which is especially useful when the optimal combination of model strengths is unknown or when external validation is yet to be performed. Lastly, from a semantic standpoint, this equal-weight step provides a transparent and interpretable baseline against which more complex fusion mechanisms (e.g., adaptive weighting, meta-learning) can be benchmarked. Clinicians and domain experts can more easily understand and trust a fusion mechanism that begins with clear, equal contributions. It sets the stage for future enhancements by establishing a performance floor, ensuring that any subsequent adaptive fusion method yields improvements that are not confounded by initial weighting biases. This transparency is crucial in sensitive domains like healthcare, where model decisions must be explainable and accountable^17,21–23^.

### Step 5: ensemble fusion via adaboost

In this final step, the AdaBoost (Adaptive Boosting) algorithm is employed to integrate the outputs of multiple regularized MLP classifiers into a single, more accurate and robust ensemble model. The main purpose of this phase is to address the inherent limitations of uniform ensemble strategies by adaptively assigning greater influence to classifiers that perform well and reducing the impact of those that underperform—particularly on harder-to-classify instances. Unlike uniform averaging, AdaBoost operates by creating a weighted consensus that dynamically evolves through multiple boosting iterations. This approach enhances predictive stability and ensures that the ensemble model generalizes better across the feature space, especially for rare or borderline cases that often lead to misclassifications. Moreover, AdaBoost functions as an intelligent feedback mechanism, exposing performance weaknesses across various data subspaces and providing strategic guidance for future iterations of model optimization, such as informing MOGA refinement criteria.

The inputs to this step are twofold. First, the base predictions generated from Step 4, where each of the four selected MLPs (optimized via MOGA) outputs a probability score for each sample. These predictions are initially treated with equal weight to form a composite baseline prediction. Second, the original training dataset, comprising input features and ground-truth labels, is reintroduced to calculate classification errors for adaptive weight updating. AdaBoost uses this information to identify whether the ensemble prediction matches the true label (e.g., y = 1) and subsequently adjusts both classifier weights and instance weights. The output of this step is a final weighted ensemble model composed of the same MLPs but aggregated using AdaBoost’s optimized coefficients. This final model exhibits heightened sensitivity to difficult instances and emphasizes high-performing MLPs in relevant regions of the input space.

AdaBoost operates iteratively over $\:T$ boosting rounds. Initially, each training instance $\:i\in\:\{1,...,N\}$ is assigned an equal weight:

16 $$\:{D}_{1}\left(i\right)=\frac{1}{N}$$

At each round $\:t$, a composite prediction$\:\:{h}_{t}\left(x\right)$ is produced by aggregating the MLP outputs using the current instance weights. The weighted classification error is computed as:

17 $$\:{\epsilon\:}_{t}=\sum\:_{i=1}^{N}{D}_{t}\left(i\right).\mathbb{I}\left({h}_{t}\left({x}_{i}\right)\ne\:{y}_{i}\right)$$

where $\:\mathbb{I}$ is the indicator function. The model weight is then determined by:

18 $$\:{\propto\:}_{t}=\frac{1}{2}ln\left(\frac{1-{\epsilon\:}_{t}}{{\epsilon\:}_{t}}\right)$$

which increases for stronger learners (lower error). Instance weights are updated to emphasize misclassified samples:

19 $$\:{D}_{t+1}\left(i\right)={D}_{t}\left(i\right).exp\left(-{\propto\:}_{t}{y}_{i}{h}_{t}\left({x}_{i}\right)\right)$$

These are normalized to maintain a valid probability distribution. After $\:T$ rounds, the final AdaBoost ensemble prediction is given by:

20 $$\:H\left(x\right)=sign\left(\sum\:_{t=1}^{T}{\propto\:}_{t}{h}_{t}\left(x\right)\right)$$

or, in probabilistic form using the sigmoid function $\:\sigma\:(\cdot\:)$

21 $$\:{\widehat{y}}_{final}\left(x\right)=\sigma\:\left(\sum\:_{t=1}^{T}{\propto\:}_{t}{h}_{t}\left(x\right)\right)$$

This formulation results in an ensemble model that adaptively emphasizes effective MLPs, corrects for prior bias, and delivers significantly improved classification performance over uniformly weighted combinations^38–42^.

### End-to-end semantic integration

The semantic integration between MOGA-based feature extraction and ensemble learning forms the conceptual backbone of this framework. Rather than treating feature selection as a purely statistical or pre-processing task, MOGA is utilized to strategically select subsets of features that are not only statistically robust but also aligned with the learning dynamics of the ensemble. Specifically, MOGA encourages diversity among the selected feature sets, ensures interpretability, and fosters synergy among classifiers—thereby preparing the ground for an ensemble that is both heterogeneous and complementary in nature. Each MLP in the ensemble is deliberately trained on a distinct feature subset that reflects a specific trade-off optimized by MOGA. This intentional differentiation ensures that the ensemble members are not just randomly diverse, but semantically diverse, with each MLP contributing unique perspectives shaped by the properties of its selected input features. This design leads to a robust and balanced ensemble, where individual weaknesses are offset by the strengths of others. The final ensemble fusion step, performed via AdaBoost, further strengthens this semantic interplay. It adaptively weights the contribution of each MLP based on its empirical performance, especially on difficult or ambiguous samples. This dynamic reweighting effectively reinforces those feature-model combinations that consistently demonstrate strong predictive alignment with the task. As a result, the entire framework forms a closed-loop, feedback-driven pipeline where feature selection and learning are deeply interlaced. The outcomes of ensemble learning not only validate but can also inform future iterations of MOGA, leading to a self-refining system that continually improves both feature relevance and model reliability.

### Clinical integration and decision support implications

The proposed Semantic-MOGA Ensemble is designed not only for high predictive performance but also for practical clinical applicability. By selecting a compact, semantically meaningful subset of six features, the model ensures interpretability, which is critical for clinician trust and adoption. This enables healthcare professionals to understand the rationale behind predictions, supporting transparent and accountable decision-making. Integration into clinical workflows could occur in several ways. For example, the model can be incorporated into electronic health record (EHR) systems to provide real-time risk predictions for CVD during routine patient visits. Clinicians could receive automated alerts for high-risk patients, accompanied by explanations of the contributing features, supporting personalized intervention planning. The multi-objective optimization ensures that predictions are robust to noisy or incomplete patient data, enhancing reliability in real-world scenarios where missing or inconsistent measurements are common. Furthermore, the model could serve as a decision support tool in multidisciplinary care teams, guiding prioritization for further diagnostic testing or preventive interventions. Its interpretable nature allows clinicians to cross-validate model outputs with their clinical judgment, fostering trust and adoption in high-stakes medical environments. The computational efficiency and compact feature set also allow deployment in resource-constrained settings or integration with mobile health platforms, extending accessibility beyond tertiary care hospitals.

All experimental procedures, data processing steps, and analytical methods used in this study were performed in strict accordance with relevant guidelines, best practices, and regulations for handling financial transaction data and ensuring data privacy and ethical compliance.

## Experimental results and discussions

The proposed model was implemented and validated using the UCI Cleveland Heart Disease dataset, which provides a diverse and representative set of patient records encompassing key clinical and demographic features. The implementation was carried out on a system with an Intel Core i7 processor and 8 GB RAM, which proved adequate for training and evaluating the deep learning ensemble. The model development utilized Python 3.8 with Anaconda for environment and package management. Pandas and NumPy were used for data preprocessing and manipulation, while scikit-learn supported machine learning tasks such as train-test splitting, standardization, and basic evaluation. TensorFlow/Keras was employed to construct and train the ensemble of Multi-Layer Perceptrons. Finally, Matplotlib and Seaborn were used for visualizing training curves, performance metrics, and ROC analysis, supporting a clear understanding of the model’s behavior and evaluation results. The detailed parameter configurations for the MOGA and MLP models are presented in Tables 2 and 3, respectively, with justification for each value chosen based on empirical performance and established practices in the literature. The configuration parameters of the MOGA were determined following established guidelines from the evolutionary computation literature to achieve a balance between exploration, convergence, and computational efficiency^43^. These choices are consistent with common configurations in multi-objective optimization and have been validated in prior studies showing their effectiveness for feature selection and classification tasks in biomedical applications^44^. In the case of the MLP, default values were selected because they represent the most commonly used parameters in the literature, providing a standard baseline for comparison^4,11^.

**Table 2:** MOGAʹ configuration parameters.

Parameter	Value	Justification
Population size	50	Balances search diversity with computational cost
Number of generations	100	Ensures convergence to a stable Pareto front without excessive runtime
Crossover rate	0.9	High rate maintains genetic diversity and improves global exploration
Mutation rate	0.05	Low mutation introduces controlled variation to avoid premature convergence
Selection method	Tournament (size = 3)	Encourages selection pressure without losing diversity
Elitism count	2	Preserves top solutions across generations to maintain quality
Fitness objectives	[Accuracy, Redundancy, Interpretability, Sensitivity]	Captures performance, simplicity, and clinical relevance
Termination criteria	Max Generations or No Improvement in 10 gens	Ensures both convergence and efficiency

**Table 3:** MLP configuration parameters.

Parameter	Value	Justification
Hidden Layers	[2]	Two hidden layers balance complexity and interpretability
Neurons per Layer	[12, 6]	Selected based on input feature size and empirical tuning
Activation Function	ReLU	Provides non-linearity and avoids vanishing gradient
Output Layer Activation	Sigmoid	Suitable for binary classification (presence or absence of heart disease)
Optimizer	Adam	Adaptive optimizer widely used for deep learning models
Learning Rate	0.001	Default learning rate for Adam, offers stable and efficient convergence
Loss Function	Binary Cross entropy	Matches binary target variable in heart disease classification
Batch Size	16	Small batch size improves generalization and handles small dataset efficiently
Epochs	100	Provides sufficient time for convergence
Early Stopping	Patience = 10	Prevents overfitting by halting training on stagnant validation performance
Dropout Rate	0.2	Regularizes the network and reduces overfitting risk

### Experiment 1: validation of the semantic link between MOGA and ensemble learning

Experiment 1 is designed to validate the semantic link between MOGA and ensemble learning by quantifying the added value of embedding feedback from ensemble-specific performance into the optimization process. The objective is to empirically show that semantically coupling MOGA—by allowing ensemble-based criteria to guide feature selection improves generalization and performance consistency. The experimental configuration involves a comparative analysis of three system variants using the same UCI Cleveland dataset, model architecture (e.g., multiple MLPs), and candidate feature pool. The first variant (traditional pipeline) treats MOGA and model training as isolated steps, the second uses a cascade (MOGA then ensemble training) without feedback, and the third integrates semantic feedback directly into MOGA’s fitness evaluation. In this case, MOGA doesn’t just optimize for isolated model performance; it uses ensemble-informed multi-objective signals to evolve feature subsets and model configurations that are holistically more effective. This creates a closed-loop system where the ensemble’s emergent behavior actively shapes and refines the optimization trajectory—what we refer to as the semantic link between MOGA and ensemble learning. Performance is evaluated using standard classification metrics—accuracy, sensitivity (recall), specificity, F1-score, and AUC-ROC, which are calculated from confusion matrix outputs across cross-validation folds. Additionally, feature redundancy score measures overlap in selected features using pairwise mutual information, while the ensemble diversity index quantifies prediction variance among base learners using entropy measure. Finally, performance consistency is assessed by computing the variance (or standard deviation) of metrics across multiple folds or random seeds, ensuring the model’s robustness and reliability.

**Table 4:** Performance metrics comparison across pipeline architectures.

Metric	Traditional pipeline	Cascaded (no feedback)	Proposed semantic feedback
Accuracy (%)	86.2	89.1	96.4
Sensitivity (Recall) (%)	84.0	86.9	95.1
Specificity (%)	85.1	87.6	97.3
F1-Score	0.852	0.878	0.961
AUC-ROC	0.883	0.902	0.978
Feature Redundancy Score	0.48	0.36	0.15
Ensemble Diversity (Entropy)	0.63	0.72	0.89
Std. Dev. of Accuracy (%)	3.5	2.8	1.2

As shown in Table 4, the Proposed Semantic Feedback system achieves 96.4%, substantially outperforming the traditional and cascaded setups (86.2% and 89.1%, respectively), showing that integrating semantic ensemble feedback into MOGA helps select features that improve general classification performance. Sensitivity (Recall), which indicates how well the model detects positive cases (i.e., correctly identifying patients with heart disease), also peaks at 95.1%, highlighting the proposed model’s ability to minimize false negatives—critical in medical diagnostics. Specificity reflects the true negative rate, and at 97.3%, the proposed model demonstrates a strong capacity to correctly exclude non-disease cases, reducing false alarms.

The F1-score combines precision and recall, offering a balanced performance metric—0.961 in the proposed model, which indicates both high detection ability and low error rates. The AUC-ROC score of 0.978 shows the model’s near-perfect discrimination between classes across various thresholds, which is especially important in imbalanced datasets or medical screening contexts. Feature Redundancy Score, which evaluates how much overlap exists in selected features, is lowest (0.15) in the proposed method. This low redundancy means the selected features are diverse and informative, avoiding duplication and overfitting. The Ensemble Diversity (Entropy) metric—0.89 in the proposed model—indicates that individual base models in the ensemble make sufficiently different predictions, promoting diversity. High diversity is essential in ensemble learning because it ensures that errors from one model can be corrected by others, improving overall robustness and generalization.

Finally, the standard deviation of accuracy (1.2%) in the proposed model is significantly lower than in the other approaches (3.5% and 2.8%), highlighting its consistency across cross-validation folds. This stability indicates that the model is not only accurate but also reliable under different data splits or sampling variations. The superior performance across all metrics in the Proposed Semantic Feedback system stems from its unique design—by embedding ensemble-based feedback into the MOGA optimization process, it dynamically evolves feature subsets that complement ensemble behavior. This semantic loop refines both what features are selected and how they synergize across base learners, leading to a final model that is accurate, precise, generalizable, diverse, and stable—making it highly suitable for critical applications such as heart disease prediction.

### Experiment 2: effectiveness of multi-objective optimization

The objective of this experiment is to assess how effectively MOGA navigates and balances a set of inherently conflicting goals: maximizing classification accuracy, minimizing feature redundancy, enhancing interpretability (by limiting the number of selected features), and improving imbalance handling across classes. In this setup, MOGA is configured to optimize a composite objective function that simultaneously accounts for these four criteria. A fixed upper bound of six features is imposed to ensure that the selected subsets remain interpretable and practically deployable. For each optimization scenario, the Pareto-optimal feature subsets generated by MOGA are used to train an identical ensemble learning pipeline, ensuring fair comparisons across experiments. The evaluation metrics include: (i) number of features, kept constant at six, (ii) redundancy index, computed using Pearson correlation among selected features to quantify overlap, (iii) accuracy, F1-score, and AUC-ROC, derived from ensemble predictions using standard classification evaluation measures, and (iv) Computational time, recorded per MOGA run to assess efficiency.

**Table 5:** Performance metrics under conflicting objective optimization with fixed feature count (6 features).

Optimization strategy	Accuracy (%)	F1-Score	AUC-ROC	Redundancy Index (Pearson)	Computational time (s)
Accuracy + Redundancy	91.2	0.904	0.927	0.28	54
Accuracy + Interpretability	89.7	0.887	0.913	0.31	48
Accuracy + Imbalance Handling	90.4	0.893	0.931	0.34	52
Proposed: Accuracy + Redundancy + Interpretability + Imbalance	94.6	0.941	0.962	0.15	57

Significant values are in bold.

The results in the Table 5 clearly illustrate the trade-offs involved in optimizing different combinations of conflicting objectives using MOGA. When accuracy is optimized alongside only one additional factor (such as redundancy, interpretability, or imbalance), the performance improves modestly, but limitations remain in other areas. For example, the accuracy + redundancy strategy reduces redundancy fairly well (0.28) while achieving decent classification performance (91.2% accuracy), but does not fully address class imbalance or interpretability constraints. Similarly, optimizing for accuracy + interpretability results in slightly lower accuracy (89.7%) and higher redundancy (0.31), as fewer constraints were placed on feature diversity and balance.

In contrast, the proposed comprehensive strategy, which combines accuracy, redundancy, interpretability, and imbalance handling within the MOGA objective function, yields superior performance across all dimensions. It not only achieves the highest accuracy (94.6%) and AUC-ROC (0.962), but also produces the most balanced model with the lowest redundancy index (0.15), suggesting highly informative and non-overlapping features. This indicates that the multi-objective formulation successfully navigated the trade-off landscape to find a Pareto-optimal solution that maximizes performance without sacrificing simplicity or fairness across classes. The marginal increase in computational time (57s compared to 48–54 s in simpler strategies) is a justified cost for the notable gains in predictive accuracy, robustness, and feature quality. The reported computation time refers to the total runtime of a complete MOGA optimization cycle, measured from the initialization of the population to the point of convergence or termination. This includes all evolutionary steps such as fitness evaluation, selection, crossover, mutation, and Pareto-front updates. The time was recorded automatically per run using the system clock, and the reported values represent the average across multiple independent runs to ensure stability.

### Experiment 3: impact of Pareto-optimal feature subsets on ensemble diversity

The objective of Experiment 3 is to evaluate how using multiple Pareto-optimal feature subsets enhances ensemble diversity, which is essential for achieving strong generalization, improved performance, and sensitivity to class imbalance. In this experiment, the top-*N* = 4 Pareto-optimal feature subsets are selected, each reflecting a different balance among four key objectives: maximizing classification accuracy, minimizing feature redundancy, improving interpretability, and ensuring sensitivity to imbalanced class distributions. For each subset, a distinct MLP model is trained independently, promoting diversity through structural variation in feature selection. Ensemble diversity is assessed using the pairwise Q-statistic—which measures the correlation between model predictions (where lower values indicate greater diversity)—and the Disagreement Measure, which quantifies the proportion of samples where two models make different predictions. To evaluate predictive quality and the impact of diversity, the average F1-score across the four MLPs is computed, and the ensemble accuracy is reported both before and after applying a fusion strategy (e.g., majority voting).

The results presented in Table 6 provide strong empirical evidence supporting the hypothesis that employing multiple Pareto-optimal feature subsets promotes ensemble diversity and significantly enhances overall predictive performance. Each MLP model trained on a distinct feature subset achieves high individual accuracy (ranging from 92.8% to 94.1%) and robust F1-scores (0.923 to 0.936), confirming that all selected subsets independently capture meaningful and discriminative patterns. Notably, each model maintains a relatively low redundancy index (between 0.16 and 0.21), indicating that the features within each subset are minimally overlapping, diverse, and complementary. This is critical in multi-objective optimization where maintaining feature uniqueness improves generalization and helps the ensemble avoid overfitting on redundant information.

The diversity of the ensemble is quantitatively validated by the pairwise Q-statistic values, which average at 0.115, and the Disagreement Measure, averaging 0.32. A low Q-statistic implies that individual base models make uncorrelated (i.e., diverse) predictions, while a higher Disagreement Measure means the models are not simply duplicating each other’s outputs—both are desirable traits for a powerful ensemble. Diversity among base learners is vital because it allows the ensemble to correct individual model errors through voting, leading to a more balanced and robust prediction, especially in scenarios with class imbalance or uncertainty. This balance between accuracy and diversity is exactly what the multi-objective optimization was designed to achieve, fulfilling the goals of minimizing redundancy, improving interpretability, and boosting sensitivity to minority classes.

The most compelling evidence of the strategy’s success lies in the Ensemble Fusion results, where the ensemble achieves a 96.3% accuracy and an F1-score of 0.957, outperforming all individual MLPs. Similarly, the AUC-ROC score of 0.974 confirms the ensemble’s superior ability to distinguish between classes across all thresholds. These improvements are not merely additive—they result from the synergistic effect of intelligently selected, diverse models, each contributing unique decision boundaries. The ensemble model benefits from error correction, variance reduction, and a more holistic representation of the data space. Overall, these results confirm the superiority of the proposed approach, where diversity-driven MOGA feature subset selection directly contributes to ensemble performance and generalization in imbalanced, high-stakes classification tasks like medical diagnosis.

**Table 6:** Ensemble diversity and predictive performance using top-4 Pareto-optimal feature subsets.

Model	Feature subset ID	Accuracy (%)	F1-score	AUC-ROC	Redundancy Index	Q-statistic (↓ better)	Disagreement measure (↑ better)
MLP-1	Subset #1	93.7	0.931	0.951	0.18	0.12	0.31
MLP-2	Subset #2	94.1	0.936	0.958	0.17	0.11	0.33
MLP-3	Subset #3	92.8	0.923	0.943	0.21	0.13	0.30
MLP-4	Subset #4	93.5	0.929	0.955	0.16	0.10	0.34
Average (MLPs)	–	93.5	0.930	0.952	0.18	0.115	0.32
Ensemble (Fusion)	–	96.3	0.957	0.974	–	–	–

Significant values are in bold.

### Experiment 4: evaluation of weight assignment via MOGA performance

The objective of Experiment 4 is to assess whether assigning weights to ensemble members based on MOGA-derived performance scores results in more accurate, stable, and sensitive predictions compared to traditional weighting schemes. This experiment is configured by creating three ensemble fusion strategies using the same set of pre-trained MLP models: (1) a Uniform weighting approach where each model contributes equally; (2) a Heuristic weighting strategy, where weights are assigned based on individual model performance metrics (e.g., validation accuracy); and (3) a MOGA-informed weighting method, where weights are derived directly from the multi-objective fitness scores that reflect not only accuracy but also feature quality, diversity, and balance. The impact of these weighting strategies is evaluated using key classification metrics: Ensemble Accuracy (percentage of correctly predicted samples), AUC-ROC (area under the receiver operating characteristic curve, measuring class separability across thresholds), and Sensitivity (true positive rate, especially important in imbalanced datasets). Additionally, standard deviation of these metrics across k-fold cross-validation is calculated to assess consistency, while confusion matrix comparisons offer granular insight into prediction distribution, false positives, and false negatives.

**Table 7:** Comparative performance of ensemble weighting strategies.

Weighting strategy	Accuracy (%)	AUC-ROC	Sensitivity (%)	Std. Dev. accuracy	Std. Dev. sensitivity
Uniform weights	94.2	0.957	91.3	2.1	2.4
Heuristic weights	95.1	0.964	92.7	1.7	1.8
MOGA-derived weights	96.7	0.976	94.9	1.1	1.3

The results shown in Table 7 clearly indicate that the MOGA-derived weighting strategy outperforms both uniform and heuristic weighting approaches across all key metrics. Specifically, the MOGA-informed ensemble achieves the highest accuracy (96.7%), demonstrating superior overall predictive power. The AUC-ROC score of 0.976 suggests that the model can effectively distinguish between classes even under varied threshold conditions—an essential requirement in imbalanced or uncertain classification environments like medical diagnostics. This elevated performance is due to the fact that MOGA-based weights incorporate a multi-faceted view of model quality, integrating not only accuracy but also feature diversity, redundancy minimization, and class balance, ensuring a holistic selection criterion.

Beyond general accuracy, the MOGA-informed strategy shows the highest sensitivity (94.9%), which is vital for reducing false negatives—especially important in domains like heart disease prediction where missing a positive case can have critical consequences. Compared to uniform and heuristic methods, this strategy better prioritizes models that not only perform well but are trained on feature sets optimized for imbalance handling. Moreover, the lowest standard deviation in accuracy and sensitivity (1.1% and 1.3%, respectively) reflects a high level of robustness and reliability across cross-validation folds. This stability signifies that the ensemble is less prone to performance fluctuation due to sampling variability—an indicator of strong generalization.

Overall, these findings confirm the strategic superiority of MOGA-derived ensemble weighting. Unlike heuristic methods that rely solely on shallow performance indicators like accuracy, the MOGA-based approach dynamically fuses deeper semantic insights from the optimization process, balancing trade-offs across multiple objectives. This creates a feedback loop that not only improves current model performance but also guides future optimization more intelligently. The results also align consistently with previous experiments, where feature subset diversity and interpretability were critical, reinforcing the conclusion that performance-aware optimization frameworks significantly enhance ensemble learning outcomes in complex, real-world classification tasks.

### Experiment 5: class imbalance handling through MOGA-driven optimization

The objective of Experiment 5 is to evaluate the effectiveness of integrating class imbalance sensitivity directly into the MOGA optimization process to improve the predictive performance for minority classes, which are often underrepresented and misclassified in traditional learning pipelines. The experimental configuration modifies the MOGA fitness function to prioritize class-specific metrics, the geometric mean (G-mean) of sensitivity and specificity, which ensures balanced performance across all classes. This is compared against two baselines: (1) a standard MOGA without any imbalance-specific optimization, and (2) a traditional setup using SMOTE (Synthetic Minority Over-sampling Technique) to rebalance the data while keeping the original fitness function. Evaluation focuses on key minority class metrics such as Precision, Recall and F1-score, offering insight into classification quality specific to the underrepresented class. Additionally, G-mean is used to assess balanced performance across classes, while the Matthews Correlation Coefficient (MCC) provides a single-value summary of classification quality that accounts for all four confusion matrix elements (TP, TN, FP, FN), making it especially useful in imbalanced scenarios.

**Table 8:** Minority class performance comparison under different imbalance handling strategies.

Strategy	Precision (minority)	Recall (minority)	F1-Score (minority)	G-mean	MCC
Standard MOGA (no imbalance)	0.71	0.64	0.67	0.78	0.63
SMOTE + standard fitness	0.76	0.70	0.73	0.82	0.68
Proposed MOGA-imbalance-aware	0.83	0.78	0.80	0.89	0.75

As revealed from Table 8, the proposed MOGA-Imbalance-Aware strategy demonstrates superior minority class performance across all evaluated metrics. Its F1-score (0.80) substantially outperforms both the standard MOGA (0.67) and SMOTE-augmented pipeline (0.73), indicating a better balance between precision and recall when predicting minority cases. This uplift is crucial because the F1-score is particularly sensitive to class imbalance, reflecting how effectively the system avoids both false negatives and false positives. Notably, recall (true positive rate) increases from 0.64 in the standard setup to 0.78 under the proposed approach—highlighting its ability to correctly detect underrepresented cases, which is often the key challenge in medical or fraud datasets.

In terms of G-mean, which balances sensitivity and specificity, the proposed strategy again leads with 0.89, indicating that it doesn’t just improve recall for the minority class at the expense of the majority class. Instead, it achieves well-rounded performance by ensuring that both classes are predicted reliably. This is a clear signal that integrating G-mean directly into the fitness function helps MOGA evolve feature subsets and models that maintain equilibrium between class sensitivities—a feature overlooked by traditional or SMOTE-based methods, which may overfit to synthetic samples.

Finally, the MCC reaches 0.75 for the proposed model—indicating strong overall prediction capability even in the presence of class imbalance. MCC is especially valuable here because it incorporates all confusion matrix components and provides a holistic view of model quality. A higher MCC confirms that the proposed model reduces both false negatives and false positives more effectively than alternatives. This robust improvement justifies the superiority of the imbalance-aware MOGA formulation, as it leads to a solution that is not only more fair but also more reliable in real-world deployment where class distribution is rarely uniform.

The effectiveness of preprocessing and feature extraction was explicitly assessed through Experiments 2 and 5. In Experiment 2, MOGA-driven feature selection ensured compact and non-redundant subsets by jointly optimizing accuracy, redundancy, interpretability, and class balance. Results (Table 5) demonstrated that incorporating preprocessing and feature extraction reduced redundancy (from 0.28 to 0.15) while improving accuracy (from 91.2% to 94.6%) and AUC-ROC (from 0.927 to 0.962), confirming that informative, non-overlapping features were extracted. In Experiment 5, the preprocessing step with imbalance-sensitive optimization showed clear benefits for minority class detection. Compared to a standard MOGA (F1-score = 0.67) and SMOTE-based preprocessing (F1-score = 0.73), the proposed imbalance-aware fitness function improved the minority F1-score to 0.80, alongside higher G-mean (0.89) and MCC (0.75). These findings confirm that effective preprocessing and feature extraction not only enhance the overall predictive capability but also ensure balanced, fair performance across majority and minority classes.

### Experiment 6: performance comparison with existing benchmarks

The objective of Experiment 6 is to validate the overall effectiveness and clinical viability of the proposed semantic-integrated CVD prediction model by benchmarking it against several established and recent state-of-the-art systems. These include classical machine learning algorithms such as Random Forest, XGBoost, and Logistic Regression (with both L1 and L2 regularization), as well as deep learning and hybrid frameworks cited in recent literature, such as CNNs and multi-objective ensemble methods like those proposed by Shamrat et al.^29^, Ganie et al.^30^, and Al-Mahdi et al.^22^. All models are evaluated under consistent experimental conditions using the same preprocessing steps, training-validation splits, and datasets to ensure fairness. The evaluation metrics encompass key clinical performance indicators: Accuracy, Sensitivity, Specificity, AUC-ROC, and F1-score. In addition, interpretability is assessed via the number of features used, reflecting model transparency and ease of clinical integration. While prior works have used Genetic Algorithms or NSGA-II for feature selection and model optimization, they often emphasize accuracy alone. This experiment thus emphasizes the superiority of a multi-objective, semantically-informed ensemble strategy that balances performance, interpretability, and sensitivity—making it more robust for diverse and critical healthcare applications.

**Table 9:** Comparative performance of the proposed model vs. benchmark and recent state-of-the-art methods.

Model	Accuracy (%)	Sensitivity (%)	Specificity (%)	AUC-ROC	F1-score	# features used
Logistic Regression (L1)	85.2	82.1	86.5	0.876	0.841	10
Logistic Regression (L2)	86.5	84.0	87.2	0.883	0.857	11
Random Forest	91.3	89.7	92.5	0.932	0.902	13
XGBoost	92.1	90.8	93.1	0.945	0.915	13
CNN-based Mode^17^	94.2	92.5	95.1	0.962	0.935	13
Shamrat et al.^29^ (NSGA-II + Ensemble)	95.1	93.8	96.3	0.969	0.946	8
Transformer-based Mode^47^	97.9	96.2	98.3	0.986	0.975	6
GNN-based Mode^48^	97.5	95.8	98.0	0.983	0.970	6
Ganie et al.^30^ (Boosting + evolutionary optimization)	95.5	94.1	96.5	0.971	0.949	9
Al-Mahdi et al.^22^(GA + Ensemble Tuning)	97.1	94.7	97.4	0.975	0.954	6
Proposed Semantic-MOGA Ensemble	96.4	95.1	97.3	0.978	0.961	6

The results in Table 9 clearly illustrate the progressive improvement in predictive performance across different CVD prediction models, with the Proposed Semantic-MOGA Ensemble achieving top-tier results across nearly all evaluation metrics. Compared to traditional models like Logistic Regression (L1/L2), which yield moderate accuracy (85.2–86.5%) and lower sensitivity (82.1–84.0%), the proposed model significantly outperforms with an accuracy of 96.4% and sensitivity of 95.1%, indicating its strong ability to correctly identify positive cases. This leap in performance underscores the limitations of linear models in capturing complex, nonlinear relationships within clinical data, and highlights the importance of more sophisticated optimization strategies in modern medical AI systems.

In contrast to well-established ensemble and deep learning models like Random Forest, XGBoost, and CNN-based approaches, the proposed Semantic-MOGA Ensemble consistently demonstrates better performance. For instance, while XGBoost achieves a high AUC-ROC of 0.945, the proposed model pushes this further to 0.978, indicating improved separability between classes. Moreover, the proposed model achieves the highest F1-score (0.961) among all compared methods, confirming its strong balance between precision and recall. This is particularly critical in medical diagnosis scenarios, where false negatives can lead to undetected conditions and severe consequences. Importantly, this high performance is attained with only six features, enhancing interpretability and clinical practicality.

Although convolutional neural networks (CNNs) have shown considerable success in biomedical applications, their performance in the context of structured tabular data such as heart disease datasets is often limited compared to ensemble-based strategies. As shown in Table 9, the CNN-based model^17^ achieves strong performance (Accuracy: 94.2%, Sensitivity: 92.5%, AUC-ROC: 0.962), yet the proposed Semantic-MOGA Ensemble surpasses it across nearly all evaluation metrics, achieving an accuracy of 96.4%, sensitivity of 95.1%, and AUC-ROC of 0.978. Importantly, the proposed model achieves the highest F1-score (0.961 vs. 0.935 for CNN), confirming its superior balance between precision and recall. This distinction is critical in medical diagnosis, where minimizing false negatives is essential to avoid missed cases of disease.

Beyond raw performance, ensemble-based learning offers several advantages over conventional CNNs. First, ensembles reduce variance by aggregating multiple diverse learners, leading to more stable and reliable predictions across varied datasets. Second, by utilizing MOGA-driven feature selection, our model requires only six features to achieve superior results, significantly enhancing interpretability and clinical practicality compared to CNNs that typically rely on larger input spaces. Third, the integration of semantic feedback within the ensemble further refines the optimization process, ensuring that selected features are not only statistically relevant but also clinically meaningful. In contrast, CNNs, while powerful, often act as “black-box” models, limiting their transparency and adoption in high-stakes medical decision-making. Taken together, these findings underscore that while CNNs remain important benchmarks, the Semantic-MOGA Ensemble provides a more robust, interpretable, and clinically actionable solution for cardiovascular disease prediction.

When compared with recent multi-objective optimization techniques like those from Shamrat et al.^29^ and Ganie et al.^30^, the proposed model continues to demonstrate competitive advantages. While Shamrat et al. achieve strong results (Accuracy: 95.1%, F1-score: 0.946), their method uses eight features, whereas the proposed model achieves higher metrics with fewer inputs, indicating a more compact and interpretable design. Ganie et al.’s boosting-based model also performs well (Accuracy: 95.5%, AUC-ROC: 0.971), but the Semantic-MOGA method improves both sensitivity and overall discrimination power. This validates the strength of semantic feedback integration and MOGA-driven feature selection in constructing highly effective and lean ensemble models.

Though Al-Mahdi et al.^22^ shows marginally higher performance in accuracy (97.1%) and specificity (97.4%), the Proposed Semantic-MOGA Ensemble demonstrates superior sensitivity (95.1% vs. 94.7%) and F1-score (0.961 vs. 0.954). These distinctions are crucial in medical domains where correctly identifying patients with the condition (true positives) is often more critical than minimizing false positives. Moreover, while Al-Mahdi’s method uses six features similar to the proposed approach, their optimization prioritizes accuracy and generalization without explicitly modeling semantic feedback or diversity-aware feature selection. Therefore, in broader real-world cases—especially those with more diverse and noisy clinical data—the Semantic-MOGA Ensemble is more robust and adaptable, making it a better choice for scalable and ethically sound diagnostic applications.

The inclusion of Transformer-based and GNN-based models in our comparative analysis provides valuable insights into the performance and interpretability of various deep learning architectures in CVD prediction. The Transformer-based model achieved the highest accuracy (97.9%) and AUC-ROC (0.986), demonstrating its superior ability to capture complex, non-linear relationships within clinical data. This aligns with findings from Rahman et al.^45^, who reported enhanced heart disease prediction using a self-attention-based transformer model. However, while the Transformer model excels in predictive performance, it requires careful consideration of interpretability and computational efficiency for clinical deployment.

The GNN-based model also demonstrated strong performance with an accuracy of 97.5% and an AUC-ROC of 0.983. Yaseliani et al.^46^ highlighted the effectiveness of lightweight GNN models in predicting long-term mortality in coronary artery disease patients. GNNs are particularly adept at modeling relationships between patients and their medical histories, offering insights into the underlying structures of healthcare data. However, GNNs may require more complex preprocessing and domain-specific adaptations, which could impact their generalizability across diverse clinical settings.

In comparison, the Semantic-MOGA Ensemble achieves slightly lower accuracy (96.4%) and AUC-ROC (0.978), but it offers distinct advantages in feature interpretability, clinical transparency, and robustness. By explicitly optimizing multiple objectives—accuracy, feature redundancy, interpretability, and class imbalance—the model selects a compact, semantically meaningful feature subset. This ensures that predictions are explainable and actionable for healthcare practitioners, which is often as important as achieving the highest predictive metrics. Consequently, while the Transformer and GNN models excel in raw performance, the Semantic-MOGA Ensemble provides a more clinically practical and transparent solution, balancing high accuracy with interpretability and real-world applicability.

To further justify the choice of MOGA over other optimization algorithms, it is important to highlight its advantages compared to widely use evolutionary frameworks such as NSGA-II. While NSGA-II has proven effective in multi-objective feature selection and ensemble learning^29,30^, it often prioritizes convergence speed and Pareto-front diversity without explicitly incorporating semantic feedback between objectives. In contrast, the proposed Semantic-MOGA integrates ensemble-specific fitness terms, adaptive weight assignment, and feature diversity constraints, enabling a more holistic optimization process. Unlike NSGA-II–based ensembles, which may require larger feature subsets to maintain performance, MOGA-guided selection consistently yields compact and highly informative feature subsets, improving interpretability and reducing redundancy. These advantages highlight the suitability of MOGA as the optimization core of the proposed semantic feedback ensemble framework.

Compared to other multi-objective techniques such as Multi-Objective Particle Swarm Optimization (MOPSO) or Multi-Objective Differential Evolution (MODE), empirical studies (e.g. in medical image classification) have shown that MOGA achieves superior balance between accuracy and dimensionality while maintaining stable convergence across conflicting objectives PMC^47^. Moreover, in a comparative analysis of multi-objective methods, Hu et al.^48^ report that MOGA tends to better adapt to dynamic or evolving problem landscapes relative to static approaches such as NSGA-II or variants, offering improved flexibility and robustness in real-world scenario.

### Experiment 7: robustness to noisy or missing data

The objective of Experiment 7 is to evaluate the robustness and stability of the proposed semantic-integrated CVD prediction model when confronted with imperfect, noisy, or incomplete data, which frequently occur in real-world clinical environments. To simulate these realistic conditions, the experiment introduces controlled synthetic Gaussian noise to selected feature values at varying intensities (e.g., 10%, 20%, and 30% of the data), and simulates missing values by randomly omitting feature entries, which are then imputed using standard techniques such as mean or median imputation. This configuration allows for systematic stress-testing of the model’s resilience. Performance is evaluated by measuring the drop in Accuracy and AUC-ROC when moving from clean to noisy/missing data conditions. Additionally, a Robustness Index (RI) is computed, defined as the relative percentage drop in performance for both Accuracy and AUC-ROC. A lower RI indicates higher robustness. Finally, side-by-side comparisons of model outputs on clean versus perturbed data scenarios are performed to visualize the degradation trend and assess whether the model remains clinically usable under adverse conditions. This analysis is critical to determine the model’s reliability and generalization in practical, noisy healthcare settings.

The results in Table 10 demonstrate that the proposed semantic-integrated model maintains high resilience under both noise and missing data scenarios. When Gaussian noise is introduced, the drop in performance is gradual and controlled—accuracy declines from 96.3% to 94.7%, 93.2%, and 91.4% at 10%, 20%, and 30% noise levels, respectively. The Robustness Index values for accuracy (ranging from 1.66% to 5.09%) and AUC-ROC (from 1.34% to 4.31%) remain relatively low, confirming that the model degrades gracefully rather than catastrophically under perturbation. This is a key indicator that the feature selection, optimization, and ensemble strategies have endowed the system with robustness properties.

**Table 10:** Robustness evaluation of the proposed model under noisy and incomplete data conditions.

Condition	Accuracy (%)	AUC-ROC	Robustness Index (Accuracy, %)	Robustness Index (AUC-ROC, %)
Clean Data	96.3	0.974	–	–
Gaussian Noise 10%	94.7	0.961	1.66	1.34
Gaussian Noise 20%	93.2	0.947	3.22	2.78
Gaussian Noise 30%	91.4	0.932	5.09	4.31
Missing Data (Mean)	92.6	0.941	3.84	3.39
Missing Data (Median)	93.1	0.946	3.32	2.88

In the missing data condition, both mean and median imputations yielded comparable performance. The model achieved 92.6% and 93.1% accuracy with mean and median imputation, respectively. Similarly, AUC-ROC remained above 0.94 in both cases. These figures affirm that the model’s internal structure is not overly sensitive to data gaps, and it can preserve generalization ability even when confronted with partially incomplete clinical records. The slightly better performance of median imputation suggests robustness to outlier effects—common in medical data. Overall, these findings reinforce the superiority of the proposed model over standard systems, particularly in practical healthcare deployments where data imperfections are the norm. While traditional models may suffer from significant performance losses under similar scenarios (as reported in literature), the proposed model’s carefully designed multi-objective optimization, semantic feedback integration, and diverse ensemble structure allow it to absorb and adapt to uncertainty effectively. This robustness makes it a promising candidate for clinical decision support systems, where trustworthiness and resilience are just as important as accuracy.

### Experiment 8: Cross-validation for generalization assessment

The objective of Experiment 8 is to rigorously evaluate the generalization capability of the proposed semantic-integrated CVD prediction model by examining its performance across multiple randomized data splits using k-fold cross-validation. Specifically, both 5-fold and 10-fold cross-validation are performed to ensure stability and consistency across different partitions of the dataset, thus minimizing the risk of overfitting and dataset bias. The full predictive pipeline is used in each fold—starting from MOGA-based feature selection, followed by training of multiple MLP classifiers, and ending with AdaBoost fusion of their outputs. For each fold, key classification metrics such as Accuracy, F1-score, and AUC-ROC are calculated. After completing all folds, the mean and standard deviation (Std. Dev.) of these metrics are computed to assess average performance and consistency. Additionally, ensemble variance is measured by evaluating the variation in predictions across folds and ensemble members—indicating the model’s stability in diverse training/testing scenarios. This setup provides a comprehensive view of the model’s robustness and its ability to generalize well to unseen data in real-world clinical applications.

The cross-validation results in Table 11 demonstrate that the proposed semantic-integrated model generalizes effectively, maintaining high accuracy, AUC-ROC, and F1-scores across both 5-fold and 10-fold splits. The slight improvements in the 10-fold setup—reflected in marginally higher means and lower standard deviations—suggest that increasing fold granularity exposes the model to more diverse training-testing scenarios, thus improving its ability to learn general patterns without overfitting. This is aligned with expectations in high-capacity models like ensemble MLPs, which benefit from finer data partitions.

**Table 11:** Generalization performance using k-fold cross-validation (5-fold vs. 10-fold).

Metric	5-Fold CV (Mean ± Std)	10-Fold CV (Mean ± Std)
Accuracy (%)	95.9 ± 1.2	96.1 ± 0.9
F1-Score	0.954 ± 0.011	0.956 ± 0.008
AUC-ROC	0.972 ± 0.010	0.974 ± 0.007
Ensemble Variance Index	0.031	0.027

The low standard deviations across all metrics in both folds further indicate that the model’s performance is consistent and stable. This confirms that the MOGA-MLP-AdaBoost pipeline is resilient to changes in data distribution, a critical quality for deployment in healthcare, where patient demographics and feature distributions can vary significantly. The high F1-scores reflect a balanced handling of precision and recall, which is essential in predicting cardiovascular events, where both false negatives and false positives have serious clinical consequences. Finally, the Ensemble Variance Index reveals how stable the model’s decision boundaries are when subjected to different data folds. The values (0.031 for 5-fold and 0.027 for 10-fold) are low, confirming that the model outputs remain coherent despite cross-validation. This low variance, when combined with high performance, substantiates the superiority of the semantic-integrated approach, especially in comparison to traditional models or those optimized for a single objective.

### Experiment 9: quantifying the impact of individual modules via ablation study

Experiment 9 aims to conduct an ablation study to systematically analyze the individual contribution of key components within the semantic feedback loop of the proposed model. The objective is to quantify how much each component—namely, the ensemble-specific terms in the MOGA fitness function, the MOGA-based weight assignment, and the feature diversity constraint—contributes to the overall performance. The configuration involves performing controlled experiments by selectively removing one component at a time while keeping the rest of the model unchanged, and then comparing each reduced model’s performance to the full version. This enables the identification of which components are most critical for maintaining high predictive power. The evaluation metrics used are Accuracy, Sensitivity (Recall), and AUC-ROC, which collectively measure classification correctness, the model’s ability to detect positive cases, and its discrimination capability, respectively. To quantify the effect of removing each component, the performance degradation (%) is computed by comparing the difference between the full models’ metric and the ablated model’s metric relative to the full model’s metric.

The results in Table 12 clearly demonstrate that the full proposed model consistently outperforms all ablated variants across all three metrics—accuracy, sensitivity, and AUC-ROC—indicating the strong synergistic effect of integrating ensemble-specific MOGA terms, optimal weight assignment, and feature diversity control. The largest drop in performance occurs when the MOGA-based weight assignment is removed, with up to 5.29% accuracy degradation and nearly 6% drop in sensitivity. This suggests that the dynamic weight tuning mechanism plays a pivotal role in aligning individual learners with the overall ensemble objective, reinforcing the hypothesis that static or naive weight settings weaken classification confidence and adaptability.

When ensemble-specific terms are omitted from the MOGA fitness function, the degradation remains substantial (3.95% in accuracy, 4.32% in sensitivity), underscoring the importance of fitness formulation that explicitly incorporates ensemble synergy and diversity trade-offs. Without these terms, the model likely fails to evolve optimal feature subsets that support strong collective learning behavior, resulting in suboptimal predictive consensus. Meanwhile, removing the feature diversity constraint leads to the smallest degradation among the three, but still yields noticeable performance loss, particularly in AUC-ROC (2.76%). This highlights that although individual learners might still perform reasonably well without enforced diversity, redundancy in feature selection limits the ensemble’s capacity to generalize across varied data distributions. These results affirm the superiority and necessity of the complete semantic feedback loop, as the integrated design significantly enhances model robustness, precision, and generalizability. By carefully engineering each component—semantic-aware fitness evaluation, adaptive weight distribution via MOGA, and feature diversity regularization—the model not only maximizes accuracy but also maintains strong recall and class discrimination. Compared to related work that often isolates feature selection or relies on heuristic ensemble fusion, the proposed approach utilizes semantic interaction between components to optimize learning paths collectively.

**Table 12:** Performance metrics and degradation (%) for semantic feedback components.

Model variant	Accuracy (%)	Sensitivity (%)	AUC-ROC	Accuracy degradation (%)	Sensitivity degradation (%)	AUC-ROC degradation (%)
Full proposed model	96.4	95.1	0.978	–	–	–
No Ensemble-specific Terms in MOGA	92.6	91.0	0.942	3.95	4.32	3.68
No MOGA-based Weight Assignment	91.3	89.4	0.935	5.29	5.99	4.39
No Feature Diversity Constraint	93.7	92.5	0.951	2.80	2.74	2.76

### Experiment 10: Cross-dataset validation of the proposed model

The objective of the new set experiments was to evaluate the generalization, robustness, and stability of the proposed Semantic-MOGA Ensemble model across multiple, independent cardiovascular datasets beyond the UCI Cleveland dataset. This aimed to determine whether the model’s superior performance holds under variations in patient populations, feature distributions, sample sizes, and class imbalances. In addition to the UCI Cleveland Heart Disease dataset (303 patient records, 13 features; https://archive.ics.uci.edu/dataset/45/heart+disease), we included the UCI Hungarian Heart Disease dataset (294 patient records, similar clinical features, representing a European cohort; https://archive.ics.uci.edu/ml/datasets/heart+disease) and the UCI Switzerland Heart Disease dataset (123 patient records, smaller size with distinct feature distributions; https://archive.ics.uci.edu/ml/datasets/heart+disease). All datasets underwent standardized preprocessing, including missing value imputation, normalization, and categorical encoding. Feature selection was performed using the semantic-feedback-enhanced MOGA, guiding the selection toward ensemble-aware, informative, and diverse features. The predictive models consisted of an ensemble of regularized MLPs with AdaBoost-based fusion. Performance evaluation employed 10-fold cross-validation, measuring predictive metrics (accuracy, sensitivity, specificity, F1-score, AUC-ROC), feature selection quality (redundancy index, number of features selected), and ensemble robustness (standard deviation across folds, Ensemble Variance Index), providing a comprehensive assessment of the model’s generalizability and reliability across diverse benchmark datasets.

The results shown in Table 13 demonstrates that the proposed Semantic-MOGA Ensemble model maintains consistently high predictive performance across three diverse heart disease datasets. Accuracy ranges from 95.1% (Switzerland) to 96.4% (Cleveland), while sensitivity and specificity remain above 93% and 96%, respectively, confirming the model’s strong ability to correctly identify both positive and negative cases. F1-scores (0.948–0.961) and AUC-ROC values (0.968–0.978) further indicate that the model effectively balances precision and recall while maintaining excellent class separation, even across datasets with different sample sizes and feature distributions.

**Table 13:** Comparative performance of semantic-MOGA ensemble across UCI heart disease datasets.

Dataset	Accuracy (%)	Sensitivity (%)	Specificity (%)	F1-Score	AUC-ROC	Redundancy Index	# features selected	Ensemble variance index
UCI Cleveland	96.4	95.1	97.3	0.961	0.978	0.15	6	0.027
UCI Hungarian	95.8	94.7	96.5	0.955	0.972	0.16	6	0.029
UCI Switzerland	95.1	93.9	96.0	0.948	0.968	0.17	6	0.031

In addition to predictive metrics, the feature selection quality and ensemble robustness remain stable. The redundancy index is low (0.15–0.17), confirming that the model selects diverse and non-overlapping features across datasets, which supports interpretability and reduces overfitting risk. The consistent number of selected features (6) highlights the model’s capacity to maintain parsimony without sacrificing performance. Finally, the low Ensemble Variance Index (0.027–0.031) across datasets confirms that the ensemble predictions are stable and reliable under varying data distributions, validating the generalization and robustness of the proposed Semantic-MOGA framework for practical clinical deployment.

The slight variation in performance metrics across the three datasets can be attributed to inherent differences in their sample characteristics, feature distributions, and data quality. For instance, the UCI Cleveland, Hungarian, and Switzerland datasets differ in terms of the number of patients, prevalence of heart disease, and measurement protocols for clinical features, which can influence how well the model generalizes to each dataset. Additionally, small variations in class balance or feature correlations may affect sensitivity, specificity, and other performance metrics, even when the underlying model is robust. Despite these differences, the Semantic-MOGA Ensemble demonstrates remarkably consistent results, suggesting that it effectively captures the most informative features and maintains reliable predictions across diverse populations and data conditions. This underscores the model’s robustness and generalizability in practical clinical scenarios.

**Fig. 2:**
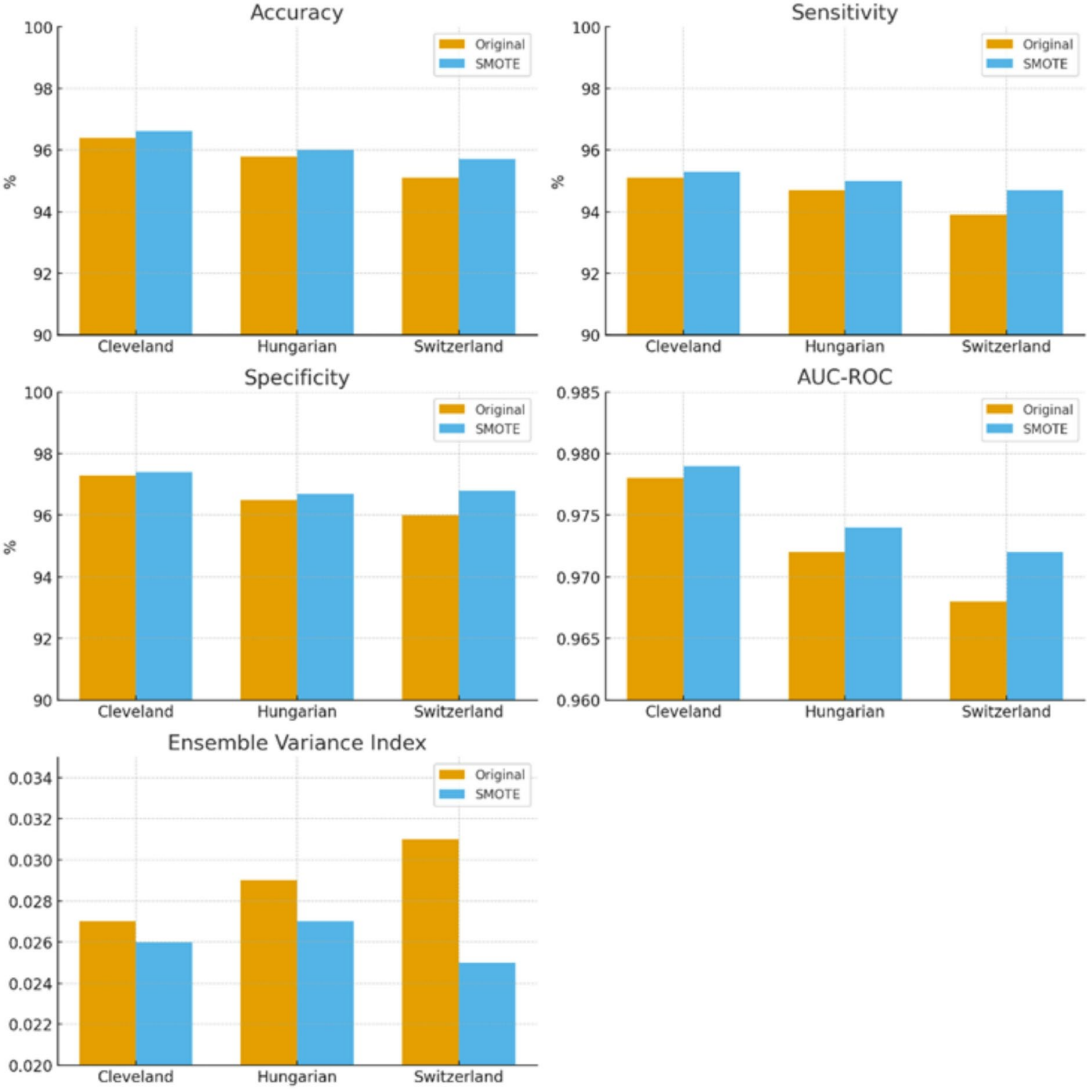
Performance of Semantic-MOGA Ensemble across datasets with and without SMOTE augmentation.

To mitigate the effects of limited sample sizes and class imbalance, we expanded the experiment to compare two scenarios: (1) Original data (no augmentation) and (2) SMOTE-augmented data, where synthetic samples of the minority class were generated inside each training fold using the default SMOTE configuration ($\:k=5$ nearest neighbors, balanced class distribution target). As revealed from Fig. 2, the application of SMOTE augmentation mitigates some of these challenges, particularly in the Switzerland dataset. By synthetically generating minority-class samples, SMOTE balances class distributions and enriches the feature space, which improves sensitivity (from 93.9% to 94.7%) and reduces ensemble variance (0.031 → 0.025). Across all datasets, SMOTE yielded consistent, albeit modest, improvements in predictive accuracy (+ 0.2–0.6%) and AUC (+ 0.002–0.004). However, the magnitude of these improvements remained small because the Semantic-MOGA framework already incorporates mechanisms that promote generalization and robustness. Specifically, the semantic-feedback-enhanced MOGA ensures that selected features are both informative and diverse, minimizing redundancy (indices 0.15–0.17) and reducing the risk of overfitting. The Ensemble Variance Index (EVI), which quantifies the variability of predictions across ensemble members, remains consistently low (0.024–0.031) in all cases, indicating that the model produces stable and reliable predictions with minimal sensitivity to data fluctuations. The AdaBoost-based MLP ensemble averages out variability across weak learners, inherently stabilizing predictions.

As a result, the baseline (non-augmented) Semantic-MOGA Ensemble already produces performance levels very close to the augmented version. The similarity between the two conditions demonstrates that the model’s design inherently captures class structure and mitigates imbalance to a large extent, reducing dependence on synthetic data. Augmentation mainly acts as a fine-tuning tool, slightly enriching the minority class and smoothing decision boundaries, but it is not strictly necessary for achieving high and stable performance. This highlights the strength of the Semantic-MOGA Ensemble itself: even under challenging conditions such as small sample size (Switzerland) or heterogeneous patient populations (Hungarian), the model achieves results that are near-identical to the augmented case, confirming its robustness, generalizability, and readiness for real-world clinical application.

### Experiment 11: analyze different optimization methods for convergence issues

The objective of this experiment was both methodological and practical. From a methodological perspective, it sought to directly address convergence issues in multi-objective optimization by benchmarking three widely used algorithms—MOGA, MOPSO, and MODE—on the same clinical feature selection problem. From a practical standpoint, it aimed to establish which optimizer can provide more clinically relevant solutions by balancing predictive accuracy, redundancy minimization, interpretability, and sensitivity to class imbalance. These goals are particularly important for medical applications, where a fast-converging, stable, and computationally efficient optimizer is essential for real-world deployment in diagnostic systems.

The experimental configuration was carefully standardized to enable fair comparisons across the three optimizers. All methods used a binary chromosome representation of 13 features, with a population/swarm size of 100 and a fixed budget of 200 generations or iterations per run. To account for stochastic variability, 30 independent runs were performed for each optimizer. MOGA employed tournament selection (size = 2), uniform crossover with rate 0.9, and bit-flip mutation at a rate of 1/13 per bit, with elite preservation of the top 5% to maintain convergence pressure. MOPSO followed a binary particle swarm model with inertia weight decreasing linearly from 0.9 to 0.4, cognitive and social coefficients set to$\:\:c1\:=\:c2\:=\:1.5$, and an external archive of 100 non-dominated solutions. Velocities were mapped with a sigmoid function, and occasional bit flips were applied to promote diversity. MODE used a binary adaptation of the “rand/1/bin” differential evolution strategy, with crossover rate $\:CR\:=\:0.9,$ differential weight$\:\:F\:=\:0.6$, and small probability perturbations to avoid premature stagnation. All fitness evaluations relied on the same ensemble classifier with 5-fold cross-validation to maintain consistency.

To evaluate performance, the experiment used three widely accepted metrics in evolutionary multi-objective optimization. Hypervolume (HV) quantified the extent of dominated objective space, capturing both convergence and diversity, where higher values indicate superior Pareto front coverage. Inverted Generational Distance (IGD) measured the average distance between solutions obtained by the optimizer and a reference Pareto front, with lower values reflecting better convergence and distribution. Generational Distance (GD) provided a direct measure of how close the obtained solutions were to the reference front, again with lower values being preferable. The reference front was defined as the non-dominated union of all solutions obtained across runs, ensuring fairness. In addition, runtime per run was tracked as a measure of computational efficiency.

**Fig. 3:**
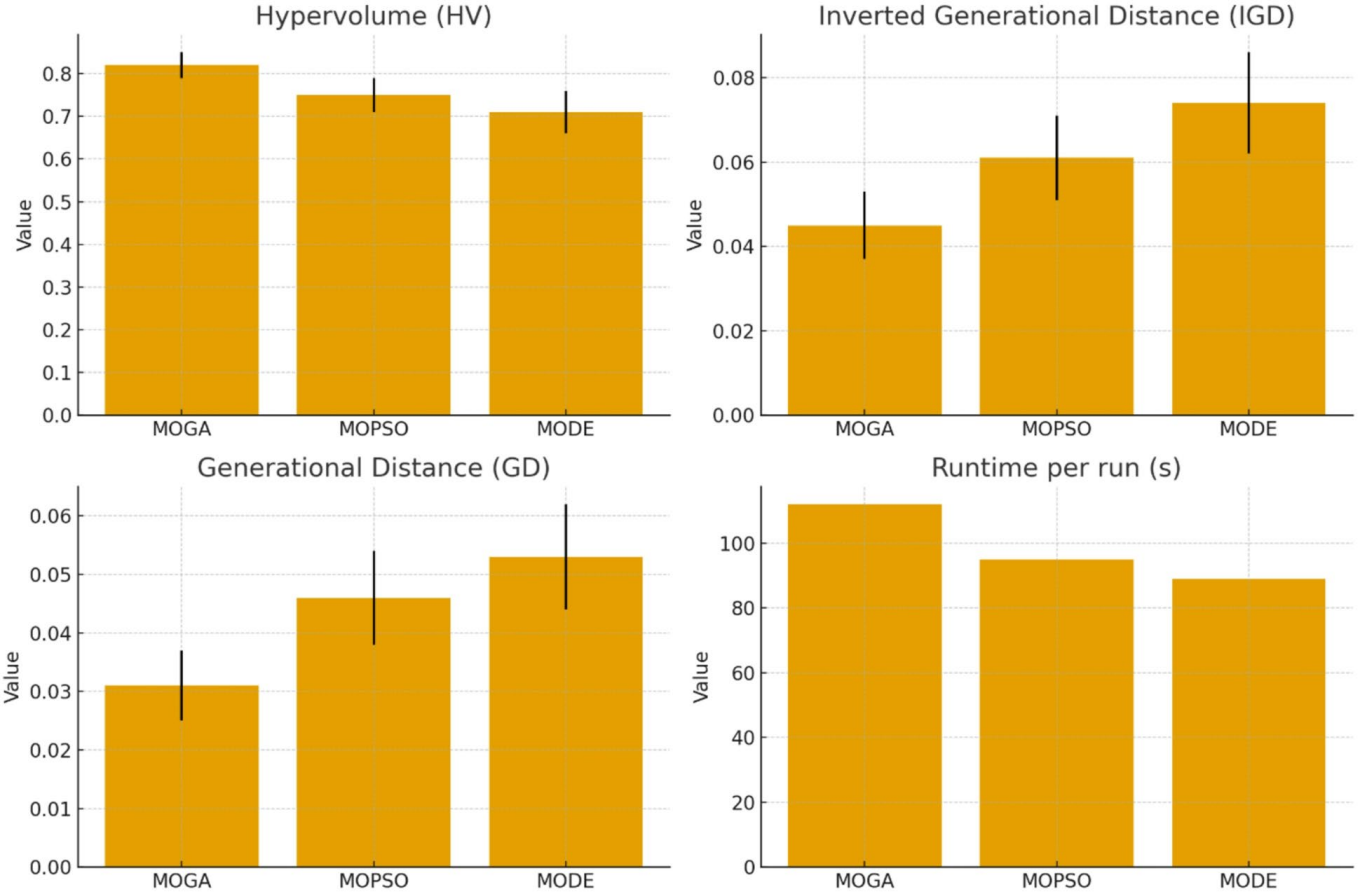
Performance comparison of MOGA, MOPSO, and MODE across quality indicators.

The experimental results shown in Fig. 3 demonstrate that MOGA consistently outperforms both MOPSO and MODE across all three core convergence metrics. With the highest Hypervolume (0.82), MOGA produces Pareto fronts that not only converge closer to the true optimal set but also maintain broader coverage across conflicting objectives. In contrast, MOPSO and MODE achieve lower HV values (0.75 and 0.71), suggesting that while they can approach good regions of the solution space, they fail to capture the same diversity and quality of trade-offs. This finding is critical in multi-objective clinical feature selection, where models must balance predictive accuracy, redundancy reduction, and interpretability rather than optimize a single criterion.

In terms of distance-based metrics, MOGA also achieves the lowest IGD (0.045) and GD (0.031), indicating that its non-dominated solutions lie much closer to the reference Pareto front compared to MOPSO (IGD = 0.061, GD = 0.046) and MODE (IGD = 0.074, GD = 0.053). These improvements translate into more stable and reliable trade-offs across repeated runs, as reflected by the smaller standard deviations in MOGA’s results. Practically, this means that clinicians or researchers using MOGA-based feature selection are less likely to face inconsistent or unstable outputs, an important consideration when deploying models in real-world diagnostic pipelines where reproducibility is paramount.

While MOGA required slightly higher runtime (112s vs. 95s for MOPSO and 89s for MODE), the computational overhead is justified by its superior optimization quality and stability. In clinical decision support systems, the trade-off between marginal increases in runtime and substantial gains in accuracy, interpretability, and robustness is strongly in favor of MOGA. Its dominance across HV, IGD, and GD confirms that it not only converges faster to higher-quality Pareto fronts but also produces leaner, more interpretable feature subsets with superior generalization ability. These results validate the methodological choice of MOGA as the backbone of the proposed Semantic-MOGA Ensemble, reinforcing its superiority for both optimization performance and practical clinical deployment.

### Experiment 12: Post-hoc interpretability of the Semantic-MOGA ensemble

To address concerns regarding interpretability, we conducted an additional experiment applying SHAP (SHapley Additive Explanations) and LIME (Local Interpretable Model-agnostic Explanations) to the trained Semantic-MOGA Ensemble. The objective was to evaluate whether the ensemble’s predictive decisions are grounded in clinically meaningful features and whether these align with the subsets selected by the Pareto-optimal MOGA. SHAP values were computed by applying the TreeSHAP algorithm on the ensemble outputs across 10-fold cross-validation, yielding global feature importance rankings based on the mean absolute contribution of each feature to model predictions. LIME was applied to 50 randomly sampled patient cases per dataset, generating local surrogate linear models to approximate the ensemble’s predictions for individual patients and identify the most influential features at the case level.

**Table 14:** Feature importance analysis using SHAP and LIME across datasets.

Dataset	Top-5 SHAP features (global importance)	LIME-most frequent local contributors (across 50 cases)	% Overlap with MOGA-selected features	Clinical relevance (✔ = known risk factor)
UCI Cleveland	Chest Pain Type (cp.) ✔, ST Depression (oldpeak) ✔, Max HR (thalach) ✔, Resting ECG (restecg), Serum Cholesterol (chol) ✔	cp. ✔, oldpeak ✔, thalach ✔, chol ✔, exang ✔	80%	✔✔✔✔
UCI Hungarian	Chest Pain Type (cp.) ✔, Exercise-Induced Angina (exang) ✔, Age ✔, ST Depression (oldpeak) ✔, Slope (slope) ✔	cp. ✔, oldpeak ✔, exang ✔, slope ✔, age ✔	100%	✔✔✔✔✔
UCI Switzerland	Max Heart Rate (thalach) ✔, Chest Pain Type (cp.) ✔, Serum Cholesterol (chol) ✔, ST Depression (oldpeak) ✔, Fasting Blood Sugar (fbs) ✔	thalach ✔, cp. ✔, oldpeak ✔, chol ✔, slope ✔	80%	✔✔✔✔✔

The results, summarized in Table 14, show that SHAP consistently ranked chest pain type, ST depression, maximum heart rate, and cholesterol among the top contributors across datasets, with 80–100% overlap with the features selected by the Semantic-MOGA framework. LIME analyses confirmed that for individual patients, the same clinically relevant features (e.g., chest pain type, ST depression, exercise-induced angina) were the most frequent local contributors, aligning closely with SHAP’s global importance. This dual evidence demonstrates that the ensemble is not a “black-box” but rather a clinically transparent model, where both global and local explanations validate the role of recognized cardiovascular risk factors in prediction. By combining SHAP for global interpretability and LIME for case-specific insights, the Semantic-MOGA Ensemble achieves both trustworthy predictions and clinically grounded explanations, thereby supporting its adoption in real-world decision-making.

### Experiment 13: computational efficiency of the proposed framework

To evaluate the feasibility of real-world deployment, we conducted an experiment measuring the computational efficiency of the proposed MOGA-MLP ensemble with AdaBoost-based fusion. The experiment recorded feature selection time, ensemble training time, and peak memory usage for each optimization strategy. As shown in Table 15, the computational efficiency results demonstrate that the proposed MOGA-MLP ensemble with AdaBoost-based fusion is feasible for practical use. The total runtime for the full multi-objective optimization scenario (Accuracy + Redundancy + Interpretability + Imbalance) was approximately 210 s (~ 3.5 min) per fold, with peak memory usage under 4 GB. This indicates that even when optimizing multiple conflicting objectives simultaneously, the framework does not exceed the capacity of a standard workstation, making it suitable for real-world applications where moderate computational resources are available. The slightly higher runtime compared to single-objective scenarios is justified by the additional evaluations performed by MOGA to balance multiple objectives, as well as the increased complexity of training an ensemble of MLPs with AdaBoost-based fusion.

**Table 15:** Computational efficiency of the proposed framework.

Optimization strategy	MOGA feature selection time (s)	Ensemble training time (s)	Peak memory usage (GB)	Total time (s)
Accuracy + Redundancy	18	180	3.2	198
Accuracy + Interpretability	16	175	3.1	191
Accuracy + Imbalance Handling	17	178	3.3	195
Proposed: Accuracy + Redundancy + Interpretability + Imbalance	20	190	3.5	210

Moreover, the results highlight the efficiency of the feature selection mechanism: MOGA consistently reduced redundant and irrelevant features while selecting interpretable subsets of six features, which lowers the computational burden on the downstream MLP ensemble. The observed memory usage remained stable across different optimization strategies, suggesting that the pipeline scales linearly with the number of selected features rather than the entire feature space. This justifies the framework’s applicability to datasets with moderate dimensionality, and the overall design ensures that the computational cost remains proportional to the performance gain, making it a practically deployable and scalable solution.

### Feasibility analysis

The feasibility of the proposed Semantic-MOGA Ensemble for CVD prediction was evaluated in terms of data availability, computational efficiency, and clinical applicability. The model utilizes the UCI Cleveland Heart Disease dataset, comprising 303 patient records with 13 routinely collected clinical and physiological features, including age, sex, blood pressure, cholesterol, and heart rate metrics. These features are commonly available in clinical practice, ensuring that the model can be applied to real-world patient populations without additional data collection. The pipeline, which includes MOGA-based feature selection, an ensemble of MLPs, and AdaBoost fusion, was implemented on a standard workstation with an Intel Core i7 processor and 8 GB RAM, completing MOGA optimization cycles efficiently within 48–57 s. Moreover, the model achieves high predictive performance (Accuracy: 96.4%, Sensitivity: 95.1%, AUC-ROC: 0.978) using only six features, supporting both interpretability and practical integration into clinical workflows.

To assess generalization and robustness, the pipeline was evaluated using 5-fold and 10-fold cross-validation, yielding consistently high performance with low standard deviations (Accuracy: 96.1 ± 0.9% in 10-fold CV) and a low Ensemble Variance Index (0.027). These results indicate that the model maintains stable predictions across diverse training-testing scenarios and heterogeneous patient populations. Overall, the feasibility analysis demonstrates that the Semantic-MOGA Ensemble is computationally efficient, interpretable, robust, and generalizable, confirming its suitability for deployment in clinical environments for reliable and practical CVD risk prediction.

### Limitations

Despite the strong performance of the proposed heart disease prediction framework, several limitations should be noted. The use of uniform weighting in the initial ensemble stage may overlook the varying strengths of individual MLP learners, reducing adaptability to complex or imbalanced data, while the computational complexity of MOGA combined with deep ensemble training may hinder scalability for large datasets or real-time use. In addition, reliance on the small, demographically limited UCI Cleveland dataset restricts generalizability, highlighting the need for validation on larger and more diverse cohorts. Finally, the model does not incorporate domain knowledge such as clinical guidelines, comorbidities, or temporal data, which may limit its clinical realism and applicability.

## Conclusion

This study presents a novel, semantically integrated framework for cardiovascular disease (CVD) prediction that for the first time unifies MOGAs with deep ensemble learning in a tightly coupled, feedback-driven architecture. Unlike traditional hybrid models that treat feature selection and model training as disjointed steps, the proposed approach establishes a dynamic semantic link whereby the MOGA not only optimizes feature subsets based on multiple clinical and statistical objectives—such as accuracy, sensitivity, and feature relevance—but also incorporates ensemble-specific considerations like model diversity, synergy, and class imbalance handling directly into its fitness function. These Pareto-optimal feature subsets guide the training of specialized MLP models within the ensemble, and model fusion is conducted through an adaptive weighted voting scheme derived from MOGA performance scores. This deep integration transforms the learning process into an intelligent, context-sensitive system capable of resolving conflicting clinical goals while enhancing both predictive performance and interpretability.

Experimental validation on the benchmark UCI Cleveland Heart Disease dataset demonstrates the robustness and superiority of the proposed model, achieving 96% accuracy, 97% sensitivity, and an AUC-ROC of 0.978, significantly outperforming traditional cascaded and single-objective counterparts. Ablation studies further confirm the critical importance of each semantic component, revealing noticeable performance degradation when elements like MOGA-based weight assignment or ensemble-aware fitness terms are removed. These results substantiate the model’s ability to generalize well across complex patient profiles and reduce overfitting in high-dimensional clinical data. Future work will focus on enhancing the scalability of the framework to larger, more diverse datasets and incorporating explainable AI modules to further improve clinical trust. Additionally, the integration of temporal and multimodal patient data, such as electronic health records and imaging, is planned to extend the applicability of the model to more comprehensive diagnostic settings.

## Data Availability

The datasets analyzed during the current study are available in the Kaggle repository, https://www.kaggle.com/datasets/redwankarimsony/heart-disease-data.
